# Phylotaxogenomics for the Reappraisal of the Genus *Roseomonas* With the Creation of Six New Genera

**DOI:** 10.3389/fmicb.2021.677842

**Published:** 2021-08-13

**Authors:** Anusha Rai, Uppada Jagadeeshwari, Gupta Deepshikha, Nandardhane Smita, Chintalapati Sasikala, Chintalapati Venkata Ramana

**Affiliations:** ^1^Department of Plant Sciences, School of Life Sciences, University of Hyderabad, Hyderabad, India; ^2^Bacterial Discovery Laboratory, Centre for Environment, Institute of Science and Technology (IST), Jawaharlal Nehru Technological (JNT) University Hyderabad, Hyderabad, India

**Keywords:** phylotaxogenomics, average amino acid Identity (AAI), percentage of conserved proteins (POCP), reclassification, *Roseomonas*

## Abstract

The genus *Roseomonas* is a significant group of bacteria which is invariably of great clinical and ecological importance. Previous studies have shown that the genus *Roseomonas* is polyphyletic in nature. Our present study focused on generating a lucid understanding of the phylogenetic framework for the re-evaluation and reclassification of the genus *Roseomonas.* Phylogenetic studies based on the 16S rRNA gene and 92 concatenated genes suggested that the genus is heterogeneous, forming seven major groups. Existing *Roseomonas* species were subjected to an array of genomic, phenotypic, and chemotaxonomic analyses in order to resolve the heterogeneity. Genomic similarity indices (*d*DDH and ANI) indicated that the members were well-defined at the species level. The Percentage of Conserved Proteins (POCP) and the average Amino Acid Identity (AAI) values between the groups of the genus *Roseomonas* and other interspersing members of the family *Acetobacteraceae* were below 65 and 70%, respectively. The pan-genome evaluation depicted that the pan-genome was an open type and the members shared 958 core genes. This claim of reclassification was equally supported by the phenotypic and chemotaxonomic differences between the groups. Thus, in this study, we propose to re-evaluate and reclassify the genus *Roseomonas* and propose six novel genera as *Pararoseomonas* gen. nov., *Falsiroseomonas* gen. nov., *Paeniroseomonas* gen. nov., *Plastoroseomonas* gen. nov., *Neoroseomonas* gen. nov., and *Pseudoroseomonas* gen. nov.

## Introduction

The genus *Roseomonas* comes under the family *Acetobacteraceae*, the class *Alphaproteobacteria*, and the phylum *Proteobacteria* (Rihs et al., 1993). Altogether, there are 46 validly published genera names under the family *Acetobacteraceae* to date.^1^ Members of the genus *Roseomonas* are metabolically diverse and prevalent (Rihs et al., 1993; Han et al., 2003). They may have fundamental functions in supporting various ecological and biogeographical processes. They are highly ubiquitous and have been widely isolated from clinical specimens like blood, wounds, and genitourinary samples (Bibashi et al., 2000; Han et al., 2003) and environmental samples like contaminated oil sediments (Subhash and Lee, 2018), water (Gallego et al., 2006), soil (Kim and Ka, 2014), and air (Kim et al., 2013). Members of this genus are aerobic, Gram-negative, pink pigmented, and non-fermentative (Rihs et al., 1993). At the time of writing, the genus *Roseomonas* comprised 45 validly described species names and two sub-species names.^2^ The genus *Roseomonas* was first described by Rihs et al. (1993). The type species of this genus is *Roseomonas gilardii* (Rihs et al., 1993) and the species name was validated in the year 1998. “*Roseomonas aceris*” (Tonouchi and Tazawa, 2014), “*Roseomonas baikalica*” (Andreeva et al., 2007), “*Roseomonas chloroacetimidivorans*” (Chu et al., 2016), and “*Candidatus* Roseomonas massiliae” (Greub et al., 2004) are not valid names. “*Roseomonas hellenica*” was described recently (Rat et al., 2021). Furthermore, *R. fauriae*, described by Rihs et al. (1998), is a later heterotypic synonym of *Azospirillum brasilense* (Tarrand et al., 1978; Helsel et al., 2006).

Phylotaxogenomics of the class *Alphaproteobacteria* was extensively studied recently by Hördt et al. (2020), which showed that several taxa under it, including the members of the family *Acetobacteraceae*, were disordered. One of the major discussions under this communication is the re-evaluation of the phylogenetic relationships between the members of the genus *Roseomonas*. This is based on the fact that the genus *Roseomonas* was distinctly polyphyletic with members like *Roseomonas stagni* and *Roseomonas lacus* interspersed in between other sister groups of *Humitalea* and *Rubritepida* in the family *Acetobacteraceae* (Hördt et al., 2020). Furthermore, the same study revealed that *Rhodovarius lipocyclicus* nested within the genus *Roseomonas* based on the constrained comprehensive tree (CCT) (Hördt et al., 2020). Likewise, a study conducted by Romano-Bertrand et al. (2016) depicted that the members of the genus *Roseomonas* were organized into seven different clades based on the 16S rRNA gene phylogeny and ecology.

Ambiguity has emerged due to the poor discriminatory power of the16S rRNA gene marker for absolute resolution at the genus/species level (Fox et al., 1992; Janda and Abbott, 2007; Olm et al., 2020). Over the decade, meaningful deductions of unresolved and longstanding phylogenetic relationships between members of the genera like *Mycobacterium* (Gupta et al., 2018), *Corallococcus* (Livingstone et al., 2018), *Roseobacter* (Wirth and Whitman, 2018), *Rhodobacter* (Suresh et al., 2019), and *Lactobacillus* (Wittouck et al., 2019) have been worked out well. This resolution has been successfully elucidated owing to the robust calculated parameters and integrated comparative genomics that included average amino acid identity (AAI), average nucleotide identity (ANI), genomic signatures, and pan-genome analysis (McInerney et al., 2017). In this study, we revisited the current status of the genus *Roseomonas* based on definite and congruent genomic evidence. Here, on the basis of phylogenomic studies like phylogenetic, pan-genomic, and taxogenomic analysis, we have proposed to re-classify the genus *Roseomonas* into *Roseomonas* genus *sensu-stricto* along with six novel genera (gen. nov.).

## Materials and Methods

### Genome Sequences and Phylogenetic Analysis

The genome sequences affiliated to the family *Acetobacteraceae*, which were available publicly at the NCBI and JGI databases, were retrieved (Supplementary Table 1). A total of 34 type strains and 28 other related strains affiliated to the genus *Roseomonas* were considered in this study (Table 1). As the genome sequence of the type species of the genus *Roseomonas* was not available at the time of writing, *R. gilardii* subsp. *rosea* was taken as a representative for the type species in this analysis.

**TABLE 1:** Genome characteristics of the members of the genus *Roseomonas* and closely related strains in the family *Acetobacteraceae.*

Organism	Strains	Size (Mbp)	G+C (mol%)	Coding sequences	N50 (bp)	L50	DDBJ/EMBL/Gen bank
**Genus *Roseomonas* Group I (*n* = 11)**							
*R. gilardii* subsp. *rosea*	ATCC BAA-691^T^	4.6	70.9	4329	257,798	6	JADY00000000
	NCTC 13290^T^	4.1	70.9	3877	2,982,497	1	UGVO00000000
	Strain DE0006	5.1	70.2	4888	366,571	6	VEIX00000000
	Strain U14-5	5.4	70.1	5120	432,8147	1	CP015583
*R. mucosa*	ATCC BAA-692^T^	4.8	70.4	4484	238,615	8	JHWD00000000
	NCTC 13291^T^	5.0	70.3	4417	4,237,410	1	UGVN00000000
	Strain AU37	4.7	70.5	4613	48,204	30	LLWF00000000
	Strain B5	4.7	70.6	4553	51,105	24	ALOX00000000
	Strain TAS13	5.0	70.0	5506	11,441	130	BDLP00000000
	Strain FDAARGOS_658	4.9	70.3	4770	4,244,047	1	CP044114
	Strain FDAARGOS_362	5.1	70.3	4919	4,180,106	1	CP024588
**Group II (*n* = 9)**							
*R. rosea*	DSM 14916^T^	5.3	70.8	4950	136,395	14	FQZF00000000
	Strain SSH11	5.2	69.7	5098	181,234	10	JAGIZB00000000
*R. pecuniae*	N75^T^	4.9	71.4	4493	200,862	9	JACIJD000000000
*R. aerilata*	DSM 19363^T^	6.4	69.7	5822	202,657	11	JONP00000000
	Strain KE2513	6.5	69.5	6613	149,208	14	RCVR00000000
	Strain SG15	5.7	70.8	5655	233,037	7	JAGIZA00000000
	Strain S9.3B	6.5	71.9	6862	123,179	14	RCZP00000000
*R. vinacea*	DSM 19362^T^	6.3	70.3	5973	192,520	9	Go0013226*
*R. harenae*	CPCC 101081^T^	5.3	68.7	5250	110,178	15	WWDL00000000
**Group III (*n* = 8)**							
*R. stagni*	DSM 19981^T^	6.2	70.6	5921	263,857	7	FOSQ00000000
*R. bella*	CQN31^T^	5.9	71.5	5486	906,722	3	QGNA00000000
	Strain AR75	6.3	70.9	5963	1,093,183	3	STGB00000000
*R. algicola*	PeD5^T^	6.6	71.0	6056	452,050	5	JAAIKB00000000
	Strain SYSU M41301	6.7	71.7	6371	561,150	4	JAERQN00000000
*R. frigidaquae*	JCM 15073^T^	6.1	70.3	5637	749,226	4	JAAVTX00000000
	Strain JCM	6.0	70.3	5787	749,226	4	JAATJR00000000
*R. selenitidurans*	BU-1^T^	5.8	71.7	5365	96,152	19	JAAVNE00000000
**Group V (*n* = 3)**							
*R. arctica*	LMG 28251^T^	4.4	69.5	4305	157,805	10	JAAEDH00000000
“*R. hellenica*”	LMG 31523^T^	7.2	69.7	7179	127,414	20	JAAGBB00000000
	Strain LMG 31524	7.2	69.7	7183	79,618	24	AAGBC00000000
**Group VI (*n* = 12)**							
*R. lacus*	CGMCC 1.3617^T^	6.4	68.7	5926	379,484	7	BMKW00000000
*R. oryzicola*	KCTC 22478^T^	5.3	71.2	5030	595,371	3	JAAVUP00000000
	LMG 31161T	5.3	71.2	5270	69,348	26	JAAEDK000000000
*R. alkaliterrae*	DSM 25895^T^	4.2	72.7	3990	301,642	5	JACIJE00000000
	LMG 31230^T^	4.3	72.6	4585	16,191	69	JAAEDJ000000000
	Strain OP-27	5.2	71.2	5742	15,335	94	JACADR00000000
	Strain PWR1	4.9	72.0	4861	172,464	9	JAGIYZ00000000
	Strain HF4	5.3	71.9	5291	274,811	7	STGD00000000
	Strain MO17	5.0	71.2	5451	11,683	107	JACADQ00000000
*R. eburnea*	LMG 31228^T^	5.8	71.2	5729	161,991	12	JAAEDL00000000
*R. terrae*	LMG 31159^T^	5.8	69.2	5729	180,609	13	JAAEDI00000000
*R. soli*	LMG 31523^T^	5.2	70.8	5309	48,334	30	JAAGBB00000000
**Group VII (*n* = 17)**							
*R. cervicalis*	ATCC 49957^T^	5.1	69.0	4199	8,459	145	ADVL00000000
	Strain JR1/69-1-13	5.1	71.5	5112	246,298	6	PDOA00000000
*R. deserti*	M3^T^	6.3	71.1	5712	22,965	79	MLCO00000000
	Strain 18066	6.3	71.1	6055	33,264	62	CACSJM00000000
*R. oryzae*	KCTC 42542^T^	4.7	69.0	4224	285,650	4	VUKA00000000
	Strain KE0001	4.5	71.4	4229	417,498	4	RCVQ00000000
*R. vastitatis*	CPCC 101021^T^	5.1	68.7	4639	164,366	7	QXGS00000000
*R. rhizosphaerae*	YW11^T^	4.7	71.9	4251	124,656	12	PDNU00000000
*R. aerophila*	NBRC 108923^T^	5.7	68.9	4903	86,979	21	JACTVA00000000
	Strain 546	4.9	70.3	4624	3,777,457	1	CP061177
	Strain 573	4.9	70.3	4634	287,631	5	JACTNG00000000
*R. ludipueritiae*	DSM 14915^T^	5.3	68.8	4966	26,901	49	JACTUZ0000000
	Strain 1311	4.7	68.9	4654	184,795	9	JACTNF00000000
	Strain 1318	4.8	68.8	4661	3,565,232	1	CP061091
*R. coralli*	M0104^T^	5.0	70.9	4458	226,823	8	SNVJ000000000
*R. wenyumeiae*	Z23^T^	6.1	68.6	5595	179,822	10	RFLX00000000
	Strain Z24	5.8	68.6	6112	21,377	74	RAQU00000000
**Other taxa**							
*Rhodovarius lipocyclicus*	CCUG 44693^T^	4.6	69.9	4316	83,545	12	JAAABL00000000
*Rubritepida flocculans*	DSM 14296^T^	3.8	73.4	3772	134,898	8	AUDH00000000
*Paracraurococcus ruber*	JCM 9931^T^	7.2	72.8	6298	22,677	81	SMOA00000000
*Dankookia rubra*	JCM 30602^T^	7.8	70.1	7355	792,44	25	SMSJ00000000
*Belnapia rosea*	CGMCC 1.10758^T^	6.0	69.7	5706	232,313	7	FMZX00000000
*Belnapia moabensis*	DSM 16746^T^	6.7	68.8	6597	89,597	24	JQKB00000000
*Siccirubricoccus deserti*	SYSU D8009^T^	6.3	69.8	5668	115,287	17	JACOMF000000000
*Humitalea rosea*	DSM 24525^T^	4.9	69.6	4631	161,053	12	QKYU00000000

*Gold IDs in the IMG Database.

For the phylogenetic analysis, 16S rRNA gene sequences of type strains of all the validly published species names, effectively but not validly published names like “*R. aceris*” R-1^T^, “*R. chloroacetimidivorans”* BUT-13^T^, and “*candidatus* Roseomonas massiliae” of the genus *Roseomonas*, were taken from the NCBI database. For *Roseomonas aquatica* and *Roseomomas fluminis*, cloned sequences were also taken to understand the phylogenetic position and confirm their status within the genus *Roseomonas*. In addition, the 16S rRNA gene sequences of representative members and cloned sequences of 11 other genera affiliated to the family of *Acetobacteraceae* were also accounted for the phylogenetic analysis. The 16S rRNA sequence of *Elioraea tepidiphila* DSM 17972^T^ was used as an outgroup. MUSCLE algorithm (Edgar, 2004) of MEGA7 was used for sequence alignments and the phylogenetic analysis of the sequences was performed using MEGA7 (Kumar et al., 2016). Distances were calculated using Kimura two-parameter in a deletion manner (Kimura, 1980). Neighbor-joining (NJ), maximum likelihood (ML), and maximum parsimony (MP) methods in the MEGA7 software were used to reconstruct phylogenetic trees. Percentage support values were obtained using a bootstrap procedure (Felsenstein, 1985).

As for the phylogenomic tree reconstruction, 62 genomes of members of the genus *Roseomonas* (34 type strains, 28 other related strains) and 135 genomes representative of 43 other genera of the family *Acetobacteraceae* (as per the NCBI database) were considered for the phylogenomic study (Supplementary Table 1). A total of 92 core genes were identified in 197 genomes and were retrieved for phylogenomic analysis using the Up-to-date Bacterial Core Gene (UBCG) tool (Na et al., 2018). The genes considered for the phylogenomic tree analysis are given in Supplementary Table 2, and *Stella humosa* DSM 5900^T^ was used as an outgroup. The RAXML-based phylogenomic tree was constructed using the concatenated sequences of the 92 core genes of 197 genomes.

### Analysis of Core and Pan-Genome

Bacterial Pan-genome Analysis (BPGA) pipeline (Chaudhari et al., 2016) was applied for the analysis of the genomic diversity of *Roseomonas*. The default parameters were set to check the conserved, accessory, and strain-specific genomic traits between the members of the genus *Roseomonas*. The same process was also carried out for each of the proposed genera (*n* ≥ 3) in this study.

### Genomic Similarity Indices

Robust parameters like average nucleotide identity (ANI) and digital DNA-DNA hybridization (*d*DDH) were computed for the precise delineation of the members of the genus *Roseomonas*. Whereas, AAI and Percentage of Conserved Proteins (POCP) values were calculated within the members of the genus *Roseomonas* as well as with the members of the other genera in the family *Acetobactereaceae*. OrthoANI tool (Yoon et al., 2017) and Genome-to-Genome Distance calculator 2.1^3^ (Auch et al., 2010) were used for calculating the ANI and *d*DDH values, respectively. AAI was calculated using the AAI calculator developed by Kostas lab^4^ (Rodriguez and Konstantinidis, 2014). POCP was calculated as described by Qin et al. (2014). The obtained AAI and POCP values were used to construct a heatmap using an online tool, Morpheus,^5^ and were clustered hierarchically based on the Euclidean distance.

### Functional and Metabolic Annotations

The identification and annotation of biosynthetic gene clusters (BCGs) for the members of the genus *Roseomonas* were carried out with the antiSMASH 5.0 web server (Blin et al., 2019) which applies the Hidden Markov Models for the identification of BCGs. For the detection, strictness level was set to “relaxed.” Genomes were submitted to the KBase online software (Arkin et al., 2018)^6^ using the default parameters to generate genomic data which were later manually analyzed to gather the group-specific gene clusters.

### Virulence Factors and Pathogen-Associated Genes

To understand the pathogenicity of the members of the genus *Roseomonas*, genomes were submitted individually to the IslandViewer 4 server (Bertelli et al., 2017) in the .gbk format where *Salmonella enterica* Serovar Typhimurium LT2 was given as the reference genome. Virulence factors were also checked by submitting the genome sequences (.fas format) to the VirulenceFinder 2.0^7^ hosted by the Center for Genomic Epidemiology (CGE) against four genera of *Listeria*, *Staphylococcus*, *Escherichia*, and *Enterococcus*. The threshold for percent identity (%ID) between input and matching gene in the database was 90% with a minimum length of 60%.

### Phenotypic and Chemotaxonomic Characters

Phenotypic and chemotaxonomic characterization of each described species for taxon delineation was obtained from the original species descriptions, Bergey’s Manual of Systematic Bacteriology (Weyant and Whitney, 2005), and from other references as specified.

## Results

### Phylogenetic Analysis Based on the 16S rRNA Gene and Genome Sequence

**FIGURE 1:**
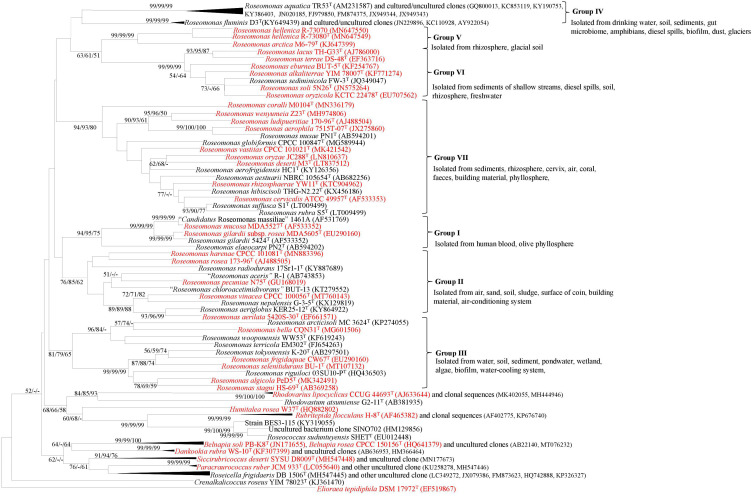
NJ phylogenetic tree based on the 16S rRNA gene sequences showing the phylogenetic relationship between the members of the genus *Roseomonas* and other closely related members of the family *Acetobacteraceae*. *Elioraea tepidiphila* DSM 17972^T^ was used as an outgroup. Numbers at nodes represent bootstrap values (given as percentages of 1,000 replications) of >50% shown at branch points (NJ/ML/ME). The GenBank accession numbers for the 16S rRNA gene sequences are shown in parentheses. Bar, 0.01 accumulated changes per nucleotide substitutions (red color indicates the availability of their genomes).

The 16S rRNA gene-based phylogenetic tree showed that the genus *Roseomonas* was polyphyletic and was segregated into seven major clades (Figure 1). These seven major clades were defined as Groups I–VII on the basis of the clade formation. The validly described members of each of the clade were as follows: Group I (*n* = 4), Group II (*n* = 8), Group III (*n* = 10), Group IV (*n* = 2), Group V (*n* = 2), Group VI (*n* = 7), and Group VII (*n* = 16) (Figure 1). Group I shall be considered as *Roseomonas* genus *sensu stricto.* The pairwise 16S rRNA gene identity was calculated using the pairwise nucleotide sequence alignment for taxonomy.^8^ The members of a group delineated by the phylogenetic tree shared at least 93.5–99.2% of the 16S rRNA gene identity (Supplementary Table 3).

**FIGURE 2:**
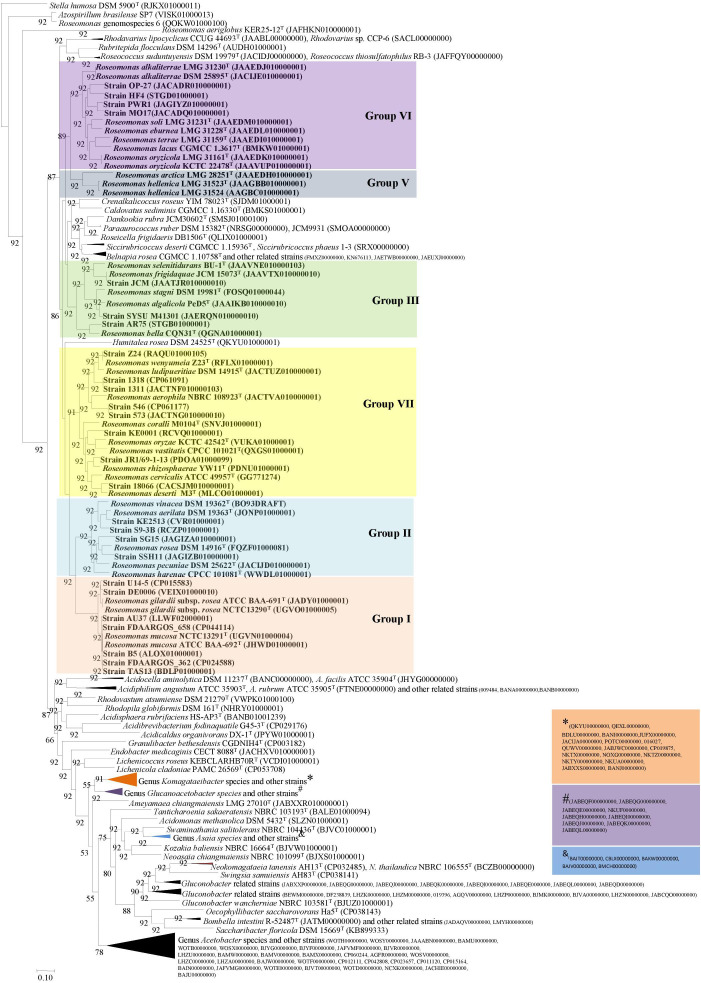
Phylogenomic tree constructed using 92 core genes tool based on the Up-to-date Bacterial Core Gene (UBCG) (Na et al., 2018). The tree was generated using the MEGA7 software (NJ) with *Stella humosa* DSM 5900^T^ (RJKX00000000) as an outgroup.

The phylogenomic tree was reconstructed using 92 core genes (Supplementary Table 2) of 197 genomes by the UBCG tool (Na et al., 2018). The study by Na et al. (2018) showed that these 92 core genes (Supplementary Table 2) were found as a single copy in more than 95% of the genome sequences. Furthermore, the UBCG-based phylogenomic tree (92 genes) could separate *E. coli/Shigella* spp. better than the 16S rRNA gene alone (Na et al., 2018). UBCG-based phylogenomic tree (Figure 2) further confirmed the heterogeneity of the genus *Roseomonas*. Genome sequences were not available for the members of Group IV including *R. aquatica* and *R. fluminis*. Furthermore, as per this study, *Roseomonas aeriglobus* and a genomospecies of *Roseomonas* cladded outside of the family *Acetobacteraceae*. Therefore, the genomes of these members were not considered for further study.

### Genomic Features of *Roseomonas* spp.

The genome information of the type strains used in this study are given in Table 1. Groups I (*n* = 11), II (*n* = 9), III (*n* = 8), V (*n* = 3), VI (*n* = 12), and VII (*n* = 17) have genome sizes of 4.1–5.4 Mbp, 4.9–6.5 Mbp, 5.8–6.7 Mbp, 4.4–7.2 Mbp, 4.2–6.4 Mbp, and 4.5–6.3 Mbp, respectively (Table 1). The genomic G+C content (mol%) of the taxa belonging to Groups I (*n* = 11), II (*n* = 9), III (*n* = 8), V (*n* = 3), VI (*n* = 12), and VII (*n* = 17) were 70.1–70.9%, 68.7–71.9%, 70.3–71.7%, 69.5–69.7%, 68.7–72.7%, and 68.6–71.9%, respectively (Table 1). On the average taxa between the groups, Group I had the lowest and Group V had the highest genome size of 4.8 and 6.3 Mbp, respectively. Similarly, the lowest (69.6 mol%) average genomic G+C content was observed with the taxa of Group V, whereas the highest (71.2 mol%) was observed with the taxa of Group VI. When the genome size of the members of the genus *Roseomonas* was compared with the taxa of the other genera in the family *Acetobacteraceae*, *Dankookia* (*n* = 1) had the highest genome size of 7.8 Mbp. On the contrary, the genus *Rubritepida* (*n* = 1) had the lowest genome size of 3.8 Mbp with the highest (73.4 mol%) genomic G+C (Table 1).

### Analysis of Core and Pan-Genome of the Genus *Roseomonas*

**FIGURE 3:**
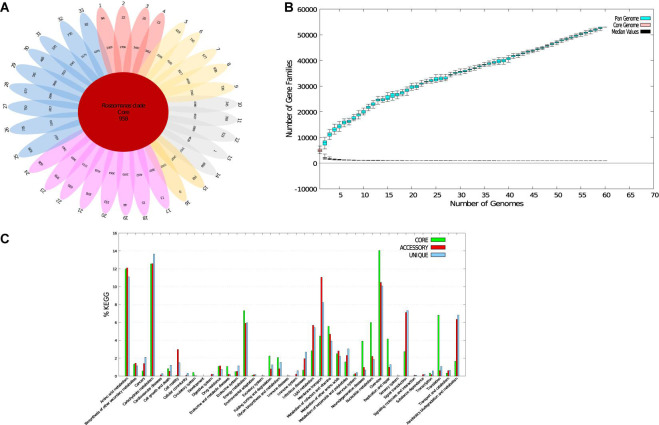
Core-pan-genome analysis of *Roseomonas* type strains. **(A)** Flower-pot diagram representing core, accessory, and unique genomes of the genomes of all strains. **(B)** Core-pan-plot for all the genomes. **(C)** KEGG distribution of core, accessory, and unique genes (1. *R. gilardi* subsp. *rosea* ATCC BAA-691^T^; 2. *R. gilardi* subsp. *rosea* NCTC 13290^T^; 3. *R. mucosa* ATCC BAA-692^T^*;* 4. *R. mucosa* NCTC 13291^T^; 5. *R. rosea* DSM 14916^ T^; 6. *R. aerilata* DSM 19363^T^; 7. *R. pecuniae* N75^T^; 8. *R.* sp. *vinacea* DSM 19362^T^; 9. *R. harenae* CPCC 101081^T^; 10. *R. stagni* DSM 19981^T^; 11. *R. algicola* PeD5^ T^; 12. *R. bella* CQN31^T^; 13. *R. frigidaquae* JCM 15073^T^; 14. *R. selenitidurans* BU-1^T^; 15. *R. arctica* LMG 28251^T^; 16. *“R. hellenica”* LMG 31523^T^; 17. *R. oryzicola* KCTC 22478^T^; 18. *R. oryzicola* LMG 31230^T^; 19. *R. alkaliterrae* DSM 25895^ T^; 20. *R. alkaliterrae* LMG 31230^T^; 21. *R. lacus* CGMCC 1.3617^T^; 22. *R. eburnea* LMG 31228^T^; 23. *R. terrae* LMG 31159^T^; 24. *R. soli* LMG 31523^T^; 25. *R. deserti* M3^T^; 26. *R. aerophila* NBRC 108923^T^; 27. *R. cervicalis* ATCC 49957^ T^; 28. *R. coralli* M0104^T^; 29. *R. ludipueritiae* DSM 14915^T^; 30. *R. oryzae* KCTC 42542^ T^; 31. *R. rhizosphaerae* YW11^T^; 32. *R. vastitatis* CPCC 101021^T;^ 33. *R. wenyumeiae* Z23^T^).

To examine the distribution of genes and genomic diversity across the genus *Roseomonas*, genome sequences were given as input in the BPGA tool. The analyzed data of the genus *Roseomonas* are given in Supplementary Table 4. Genus *Roseomonas* members have 958 core genes (19.1%), 219,753 accessory genes (72.8%), and 24,320 strain-specific genes (8.1%) (Figure 3A). For the diagrammatic representation of the pan-genome for the genus *Roseomonas*, only the type strains were considered. The core-pan plot (Figure 3B) showed an open pan-genome for the genus *Roseomonas* as it did not level off into a plateau and extended with the increase in the number of genomes. The core genome was conserved at the genus level as the plot leveled off. The KEGG distribution (%) of genes based on function is illustrated in Figure 3C for the genus *Roseomonas*. Members of Groups I, II, III, V, VI, and VII had core genes between the range of 1,573–3,152 (Supplementary Table 4). The diagrammatic representation of the core and pan genome plot for each of the group are given in Supplementary Figures 1A–F. For each of the group, the KEGG distribution (%) of genes based on function is illustrated in Supplementary Figures 2A–F.

### Genomic Metrics—dDDH, ANI, AAI, and POCP

At an intra-group level, members of a group (consisting both type and non-type strains) defined by phylotaxogenomics shared *d*DDH and ANI values of at least 21–100% and 78.0–99.9%, respectively (Supplementary Table 5). At an inter-group level, members of different groups did not show *d*DDH and ANI values above 20.8 and 76.8%, respectively. POCP and AAI values were calculated between the members of the groups (intra-group) as well as at the inter-group level (Supplementary Tables 6, 7) as they are considered pivotal and accurate for genus delineation (Qin et al., 2014). POCP values for Group I (*n* = 11), Group II (*n* = 9), Group III (*n* = 8), Group V (*n* = 3), Group VI (*n* = 12), and Group VII (*n* = 17) taxa were 80–98.5%, 65.2–71.7%, 66.9–98.7%, 65.8–99.3%, 66.4–99.2%, and 65.2–99.4%, respectively. While the AAI values were 91.9–99.0%, 76.7–84.8%, 71.8–99.9%, 73.8–99.9%, 77.0–99.9%, and 70.2–99.9% for the taxa of the respective groups. The POCP and AAI heatmaps (Supplementary Figures 3, 4) support the division of the six groups. The AAI values between *Roseomonas fauriae* (later heterotypic synonym of *Azospirillum brasilense*) and the members of Group I are in between 51.2 and 51.5%, thus making them distinct species under different genera.

### Functional and Metabolic Annotations

The BCGs prediction based on the antiSMASH 5.0 web server indicated an average *Roseomonas* genome consisted of seven BCGs (420 BCGs were found in 60 genomes). Group I (*n* = 11) members had 65 BCGs (clustered into 11 types of BCG families); Group II (*n* = 9), 72 (19 BCGs families); Group III (*n* = 8), 59 (13 BCG families); Group V (*n* = 3), 35 (9 BCG families); Group VI (*n* = 12), 68 (14 BCG families); and Group VII (*n* = 17), 124 (24 BCG families) (Supplementary Table 8). Terpenes were the most prevalent ones (two per genome on an average) but also an average of one type1 polyketide synthases (TPKS) per genome. The members of Group I exclusively had non-ribosomal peptide synthetases (NRPS), beta-lactone (fengymycin), and thioamitides (asukamycin). The members of Groups I and II both had phosphonate/terpene (phosphinothricintripeptide) BCG. All the members of Group II possessed a redox-cofactor BCG, whereas the members of both Groups II and III shared type 3 polyketide synthases (T3PKS). Group V members had NRPS/T1PKS and arylpolene BCGs. As for Group VI, eight genomes (out of 17) had NRPS and five genomes had arylpolene (xanthomonadin) BCGs. As for the Group VII, four genomes had unique ribosomally synthesized and post-translationally modified peptides (RiPPs) BCG exclusively.

The genome annotation showed that 226, 58, and 82 gene clusters were unique to Groups I, II, and III, respectively (Supplementary Table 9). Members of Group V comprised of 67 unique gene clusters, Group VI consisted of 74 unique gene clusters, and Group VII comprised16 unique gene clusters (Supplementary Table 9). As predicted by the IslandViewer 4 server and VirulenceFinder 2.0, the draft genomes of the members of the genus *Roseomonas* did not possess any genes related to its pathogenicity and virulence.

### Phenotypic and Chemotaxonomic Characters Between the Groups

Correlation between the genomic studies and phenotypic characters are desirable for supporting taxa delineation. Members of Groups II and VI can be distinguished from those of Group V by being non-motile. Members of Groups II and III can be differentiated from those of Groups I, IV, V, VI, and VII in showing variable catalase activity. Members of Groups I and II show variable oxidase activity, whereas members of the other groups are either oxidase positive (Groups III, VI, VII) or negative (Groups IV, V). Members of Groups VI and VII have a high NaCl tolerance than members of the other groups. NaCl tolerance was used as a differentiating taxonomic character for delineating the genus *Swaminathania* biochemically from the other genera of the family *Acetobacteraceae* like *Acetobacter*, *Gluconobacter*, and *Kozakia* (Loganathan and Nair, 2004). Members of Groups II and III were variable for starch hydrolysis, while those of Groups IV, V, VI, and VII were negative. Members of Group VI were variably positive for casein hydrolysis, whereas members of the rest of the groups were negative. Members of Groups I, IV, and VI were urease positive, whereas members of Groups II, III, and VII were variable in reaction. Members of Groups II, III, V, and VII showed a negative reaction for nitrate reduction, whereas those of the other groups were variable. Similarly, Groups II and III can be differentiated from the other groups in showing variable activity of gelatin hydrolysis. Group VII can be distinguished from Groups II, III, and IV in the positive utilization of D-glucose. Members of Groups V, VI, and VII could utilize L-arabinose for growth, whereas members of Groups II, III, and IV could not utilize the same. Members of Groups III, VI, and VII could utilize sucrose, whereas those of Groups IV and V were negative. Members of Group VI contained Q-9 as the unique respiratory quinone which differs from those of the other groups which contained Q-10. Groups III and VII members can be distinguished from those of Groups I, II, IV, V, and VI in having a glycolipid. Similarly, Groups V and VII can be distinguished from each other in having an unidentified lipid as one of the polar lipids. Analysis of polar lipids is a significant chemotaxonomic aid and has often been used as a differential character for reclassification. The phenotypic differentiating characteristics between the genus *Roseomonas* and other closely related genera of the family *Acetobacteraceae* are given in Table 2.

**TABLE 2:** Characteristics differentiating the *Roseomonas* groups from other closely related genera in the family *Acetobacteraceae*.

	1	2	3	4	5	6	7	8	9	10	11	12	13	14	15	16
Cell shape	Coccoid–rod	Coccoid–short rod	Cocci–rod	Coccoid–ovoid	Rod	Cocci to short rods	Coccoid–oval	Rod	Coccoid	Short rod	Coccoid	Coccoid	Coccoid	Ovoid to coccoid	Short rods	Coccoid
Cell size (μm)	0.9–1.0 × 1.9	0.8–1.2 × 1.0–1.2	0.5–1 × 0.5–2.0	0.7–1.2 × 1.2–2.0	0.7–0.9 × 1.9–3.2	0.5–1 × 0.8–2	0.6–1 × 1–3.5	0.9 × 61.4–1.7	0.7–2	1.4–1.7 × 0.7–1.2	2	0.8–1.5	0.8–1	0.6–0.9	0.8–1.2 × 1.0–1.6	0.6–0.9
Motility	v	–	v	V	+	–	v	–	ND	+	–	–	–	–	–	–
Oxidase/catalase	v/+	v/v	+/v	−/+	−/+	+/+	+/+	+/+	+/+	+/+	+/+	+/+	+/+	+/+	+/+	+/+
Optimum temp. °C (range)	35 (12–42)	25–30 (5–45)	30–37 (4–45)	28–35 (15–40)	4–45 (15–30)	40–50 (5–55)	28–35 (4–45)	30 (1–30)	25 (4–37)	50 (25–50)	28 (20–40)	30–32 (20–42)	30 (20–37)	40–50 (20–60)	30 (20–36)	4–45 (28–37)
Optimum pH (range)	6–7 (5–8)	6.5–9 (6–10)	7–8 (5–10)	7 (5–9)	4–11(6–8)	7–10 (5.5–11)	7–7.5 (5–8.5)	7 (8–9)	7 (5–8)	7.5 (7.5–8)	7 (6–8)	ND (6.6–6.8)	7 (5.5–8.5)	8 (6–10)	6 (5.5–8)	7 (4–8)
NaCl (%) toleration	0–3	0–1.02	0–2	0–2	0–2	0–6.5	0–6	0	0–3	1–2	0–2	0–4	0–0.5	0–2.5	0–1	0–1.5
**Hydrolysis of:**																
Starch	ND	v	v	–	–	–	–	ND	–	–	–	+	–	–	ND	–
Casein	ND	–	–	–	–	v	–	ND	–	–	–	–	–	+	ND	ND
Gelatin	–	v	v	–	–	–	–	–	–	+	–	–	+	+	ND	+
Urease	+	v	v	+	+	+	v	+	v	+	–	+	–	+	v	+
Tween 80	ND	v	–	–	v	–	–	+	ND	–	+	–	–	–	ND	–
Nitrate reduction/H_2_S production	v/v	–/v	–/v	v/–	–/–	v/ND	–/–	–/–	+/–	ND	ND	+/+	+/ND	+/–	ND/ND	+/–
**Utilization of:**																
D-Glucose	v	–	–	–	v	v	+	+	–	+	–	+	+	+	–	+
D-Fructose	v	–	v	–	ND	v	+	ND	–	–	–	+	ND	–	–	+
D-Galactose	v	–	–	–	ND	v	v	ND	–	–	–	+	ND	+	–	ND
D-Lactose	v	–	–	–	ND	–	v	–	+	ND	–	+	ND	+	ND	–
Sucrose	v	v	+	–	–	+	+	–	–	+	ND	–	ND	+	–	–
D-Mannitol	v	–	–	–	–	v	v	ND	–	ND	–	–	–	–	–	+
L-Arabinose	v	–	–	v	+	+	+	+	–	ND	+	+	–	–	–	–
Isoprenoid quinones	Q10	Q10	Q10	Q10	Q9	Q10	Q10	ND	Q9	Q9	Q10	Q10	Q10	Q10	Q10	Q10
Polar lipid	DPG, PE, PC, AL, PL	DPG, PE, PC, AL, PL	DPG, PE, PC, AL, GL, PL	DPG,PC, PE	DPG, PE, PC, AL, L	DPG, PE, Al, L, PL	DPG, PC, PE, GL, PL, AL, L	DPG, PE, PC, AL	DPG, PC	PC, PE, DPG, AL	PE, PL, AL	ND	PE, PL, AL	DPG, PE, PC	PE, PDME, PC, AL	DPG, PC, PE
Major FA	C_16:0,_ C_18:1_ 2-OH, C_19:__0_*_*c*__*yclo*_* ω7*c*	Summed feature 3 C_18:1_ 2-OH, C_18:1_ 3-OH, C_16:0,_ C_18:0_	Summed feature 3 C_16:__1_ *ω*5c C_16:0_	Summed feature 3 C_16:0_ C_18:1_ *ω*7c, C_18:1_ 2-OH	Summed feature 3 C_18:1_ *ω*7c C_16:0_	Summed feature 3, C_16:0_ C_18:1_ 2 O_*H*_	Summed feature 3 C_16:0_ C_18:1_ 2 OH	Summed feature 3	C_16:0_	C_16:0_	C_18:1_2-OH	C_18:1_ C_18:1_2-OH	C_18:1_2-OH	C_16:0,_ summed feature 4	C_18:__1c__*yclo*_ *ω7c*	C_16:0_

*All genera are Gram-negative; contains PG as Polar lipid; contains C_16:1_ω7c/C_16:1_ω6c as a major fatty acid; Summed features represent two or three fatty acids that cannot be separated by gas chromatography with the MIDI system. Summed feature 3, C_16:1_ω7c and/or iso-C_15:0_ 2-OH; summed feature 4, anteiso-C_17:1_ B and/or iso-C_17:1_ I. DPG, diphosphatidylglycerol; PG, phosphatidylglycerol, PE, phosphatidylethanolamine; PC, phosphatidylcholine; GL, unidentified glycolipid; PL, unidentified phospholipid; AL, unidentified amino lipids, L, unidentified Lipid; FA; Fatty acid; ND, Not determined; v, variable; +, positive, -, negative; v/ND, variable or not determined; -/v, absent/variable; +/+, positive for both tests; v/+, variable/positive; v/v, both tests show variable results; +/+, both testes positive. 1. Group I (Roseomonas genus sensu-stricto) (data based on R. gilardii 5424^T^, R. gilardii subsp. gilardii ATCC49956^T^, R. gilardii subsp. rosea MDA5605^T^, R. mucosa MDA5527^T^) (Han et al., 2003; Rihs et al., 1993); 2. Group II (data based on R. pecuniae N75^T^, R. aerilata 5420S-30^T^, R. nepalensis G-3-5^T^, R. rosea DSM 14916^T^) (Yoo et al., 2008; Sánchez-Porro et al., 2009; Lopes et al., 2011; Chaudhary and Kim, 2017); 3. Group III (R. stagni HS-69^T^, R. frigidaquae CW67^T^, R. bella CQN31^T^, R. algicola PeD5^T^, R. selenitidurans BU-1^T^) (Furuhata et al., 2008; Kim et al., 2009, 2020; Hou et al., 2020; Zhang et al., 2020); 4. Group IV (data based on R. aquatica TR5^T^, R. fluminis D3^T^) (Gallego et al., 2006); 5. Group V (based on R. arctica M6-79^T^, R. hellenica R-73070^T^) (Qiu et al., 2016; Rat et al., 2021); 6. Group VI (based on R. oryzicola KCTC 32190^T^, R. lacus JCM 13283^T^, R. alkaliterrae YIM 78007^T^) (Jiang et al., 2006; Dong et al., 2014); 7. Group VII (data based on R. deserti M3^T^, R. cervicalis KACC 11686^T^, R. oryzae JC288^T^, R. rhizosphaerae KACC 17225^T^, R. aestuarii JC17^T^) (Rihs et al., 1993; Venkata Ramana et al., 2010; Chen et al., 2014; Ramaprasad et al., 2015; Subhash and Lee, 2018); 8. Humitalea (data based on H. rosea W37^T^) (Margesin and Zhang, 2013); 9. Belnapia (based on B. moabensis CP2C^T^, B. rosea CGMCC1.10758^T^, B. soli PB-K8^T^) (Reddy et al., 2006; Jin et al., 2012, 2013);10. Rubritepida (based on R. flocculans DSM 14296^T^) (Alarico et al., 2002); 11. Dankookia (based on D. rubra WS-10^T^) (Kim W. H. et al., 2016); 12. Paracraurococcus (data based on P. ruber NS89^T^) (Saitoh et al., 1998; Alarico et al., 2002); 13. Roseicella (based on R. frigidaeris DB1506^T^) (Khan et al., 2019); 14. Crenalkalicoccus (based on C. roseus YIM 78023^T^) (Ming et al., 2016); 15. Rhodovarius (based on R. lipocyclicus CCUG 44693^T^, R. crocodyli CCP-6^T^) (Kämpfer et al., 2004; Chen et al., 2020); 16. Siccirubricoccus (based on S. deserti) (Yang et al., 2017).*

## Discussion

Genus *Roseomonas* has been widely studied as the members have clinical and environmental significances (Rihs et al., 1993; Han et al., 2003; Romano-Bertrand et al., 2016; Subhash et al., 2016). Six novel species names were validly published in the years 2020–2021 (see text footnote 2). The supremacy of the genomic era has identified whole-genome sequence data being more definitive and conclusive for taxa delineation than the 16S rRNA gene marker. Conventionally, a polyphasic approach was adopted for the demarcation of the *Roseomonas* species. As per the work conducted by Romano-Bertrand et al. (2016), they showed that the genus *Roseomonas* was segregated into seven different clades based mainly on the 16S rRNA gene phylogeny and ecology. Likewise, the study carried out by Hördt et al. (2020) showed that the genus *Roseomonas* was polyphyletic and consequently called in for a major reclassification. Taxogenomics is considered more precise and superior for the taxa delineation as it strengthens data reproducibility and reliability (Chun and Rainey, 2014; Lalucat et al., 2020). To determine the absolute phylogenetic standing between the current members of genus *Roseomonas*, a comprehensive taxogenomic analysis was carried out.

The 16S rRNA gene-based phylogenetic tree showed that the genus *Roseomonas* was polyphyletic and was segregated into seven major clades: Groups I to VII (Figure 1). The cladding patterns of Groups I, II, III, VI, and VII members in this study are comparable to the cladding pattern observed by Romano-Bertrand et al. (2016) in terms of the species composition. Group IV member, *R. aquatica* (Group IV), cladded outside the designated clade observed by Romano-Bertrand et al. (2016) whereas, *R. fluminis* which also belongs to this group was described later in 2018 by Ko et al. (2018). Furthermore, *R. arctica* (Group V) was shown to be affiliated to the *R. stagni* (Group III) clade, whereas “*R. hellenica*” is not included by them as it was described later (Rat et al., 2021). It was suggested that the whole genome phylogenies could provide a better resolution rather than a single gene like the 16S rRNA gene-based phylogenies for taxonomic delineation (Logan et al., 2009). However, in the case of *Roseomonas*, the phylogenomic tree based on 92 concatenated genes also showed the same cladding pattern (Figure 2; except for Group IV for which the genome sequences are not available).

Nouioui et al. (2018) and Hördt et al. (2020) have shown that genome size variation can be applied as a reliable taxonomic marker. They showed that the genome size appeared to be genus-specific for the members of the phylum *Actinobacteria* and the class *Alphaproteobacteria.* Both studies implied that the genome size and G+C content (mol%) were phylogenetically conserved. Hence, the genome sizes have been formally added to the descriptions. In this context, Group I had the smallest genome size as compared to the other groups. Analysis of pan and core genomes using the BPGA pipeline illustrated that the pan-genome of the genus *Roseomonas* is open and consists of 958 core genes, summing up to 19.1% of the total pan-genome (Supplementary Table 3). As for the BCGs, only groups of the genus *Roseomonas* could produce specific secondary metabolites, i.e., NRPS, fengymycin, and asukamycic (Group I); redox-cofactor (Group II); and NRPS/T1PKS and arylpolene (Group V) (Supplementary Table 8). The genome annotation also revealed the composition of the gene clusters unique to each group with the highest number of clusters unique to Group I and lowest in Group VII (Supplementary Table 9).

**FIGURE 4:**
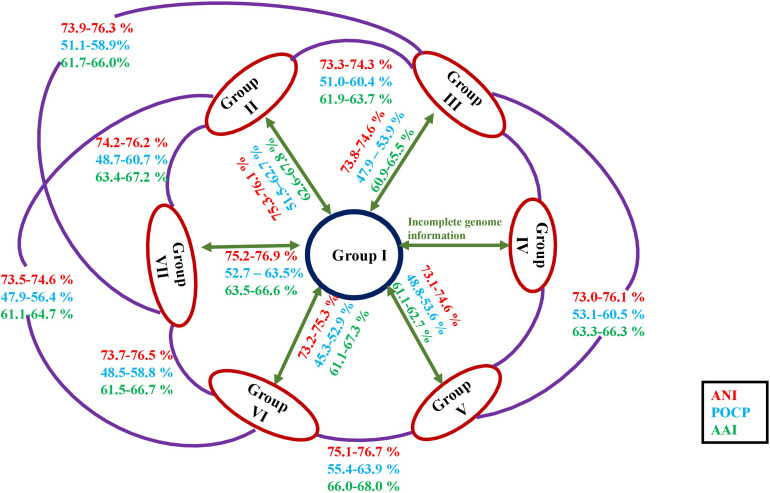
Diagrammatic representation of the ANI, AAI, and POCP values in between the members of each group of the genus *Roseomonas*.

Chun et al. (2018) have proposed minimum standards based on an overall genome-related index (OGRI) like *d*DDH and ANI for species delineation. Wayne et al. (1984) prescribed a cut-off of 70% *d*DDH value for species delineation. In the present scenario, ≥95% of ANI between the two strains concludes that both belong to the same species whereas for values <95%, two strains are considered as different species (<75% for different genera) (Richter and Rosselló-Móra, 2009; Rodriguez and Konstantinidis, 2014; Rosselló-Móra and Amann, 2015). Both indices (*d*DDH and ANI) are consistent at the intra-group level with the recommended standards confirming that all members of *Roseomonas* are well-described at the species level (Supplementary Table 3). For the POCP values, <50% were considered a cut-off for genera delineation (Qin et al., 2014). POCP values between the groups of the genus *Roseomonas* were <65% (40–65%) (Figure 4 and Supplementary Table 6) and with the other genera of the members of the family *Acetobacteraceae* were also <70% (20–60%) (Supplementary Table 6). Thus, for the species of the genus *Roseomonas*, the calculated values of POCP for genus delineation were not in agreement with the ones observed by Qin et al. (2014). Although a proposed genus boundary of the POCP value for prokaryotic lineages was assigned as 50%, many studies later showed exceptions at the inter-genera comparison. The universal cut-off of 50% is considered conservative, as being only an index of relatedness (Liu et al., 2020) as reflected in its ineffectiveness in delineating different genera of the family *Methylococcaceae* (Orata et al., 2018), *Bacillaceae* (Aliyu et al., 2016), *Burkholderiaceae* (Lopes-Santos et al., 2017), and *Rhodobacteraceae* (Wirth and Whitman, 2018). Luo et al. (2014) have shown that AAI values of related but different genera ranged from 60 to 80%. In our study, AAI values were below 70% between the different groups of the genus *Roseomonas* (Figure 4 and Supplementary Table 7) and other genera members of the family *Acetobacteraceae* (Supplementary Table 7), hence in congruence with the work of Luo et al. (2014).

The study conducted by Hördt et al. (2020) showed that certain members of the genus *Roseomonas* like *R. stagni* were interspersed with *H. rosea* and *R. lacus* with *Rubritepida flocculans.* The distinction between the mentioned species pairs was clarified by the *d*DDH, ANI, AAI, and POCP values. *d*DDH, ANI, AAI, and POCP values between *R. stagni* and *H. rosea* and *R.* lacus and *R. flocculans* were 19.4, 74.1, 64.2, and 64.2 and 19.8, 74.8, 66.6, and 57.1%, respectively. Similarly, the study also showed that *Rhodovarius lipocyclicus* was nested within the genus *Roseomonas* (Hördt et al., 2020). However, *R. lipocyclicus* is a distinct member of the genus *Rhodovarius* from *Roseomonas* as the AAI and POCP values are 50.8–66.8% and 53.2–64.7%, respectively, below the recommended cut-off for genus delineation. Thus, the above indices clearly differentiate the *Roseomonas* species from the genera of *Humitalea*, *Rubritepida*, and *Rhodovarius*. Therefore, the above detailed discussion entails the formation of seven different groups within the genus *Roseomonas* in congruence with the findings given by Romano-Bertrand et al. (2016) and Hördt et al. (2020).

Members of the genus *Roseomonas* have a ubiquitous distribution in the environment. Group I members were the only ones to have been isolated from a human blood sample (*R. gilardii*, *R. mucosa*, R. *gilardii* subsp. *rosea*) (Rihs et al., 1993; Han et al., 2003) except for *R. elaeocarpi* which was isolated from olive phyllosphere (Damtab et al., 2016). An examination of the literature demonstrated that the strains of *Roseomonas* have been widely reported in human infections and isolated from clinical samples like sputum, wounds, and genitourinary sites (Wallace et al., 1990; Rihs et al., 1993). It was also found to be associated with immunocompromised patients (Marin et al., 2001). Other reports of bacteremia caused by the *Roseomonas* species were in vertebral osteomyelitis (Nahass et al., 1995), infected tooth (Diesendorf et al., 2017), endocarditis (Shao et al., 2019), and peritonitis (Malini et al., 2016). However, many reports in case studies suggests that the majority of the patients had underlying diseases, malignancy being the most common factor (Wang et al., 2012; Michon et al., 2014). Pathogenic genes and virulence factors are potent segments of the genomic islands. They become part of the genome as a consequence of horizontal gene transfer, and these genes are observed to confer their pathogenicity and virulence to the bacteria (da Silva Filho et al., 2018). The above-mentioned reports can be substantiated with the predicted facts that the genomes of the members do not suggest any kind of genes responsible toward its pathogenicity and virulence. Thus, it may be concluded that *Roseomonas* strains are incidental and not causative of pathogenicity. In addition to being associated with human clinical samples, *Roseomonas* spp. were also isolated from various sources like soil (Kim and Ka, 2014), sediment (He et al., 2014), sludges (Wang et al., 2016), contaminated soil (Subhash and Lee, 2018), freshwater (Baik et al., 2012), and also human sources like blood (Rihs et al., 1993; Figure 1).

In an ecological context, the presence of accessory genes in the members shows their divergence due to environmental adaptations. The presence of accessory genes shows the acquisition of genes in response to the selective pressure (Brito et al., 2015) or for the colonization of the new ecological habitats (McInerney et al., 2017). The cosmopolitan distribution of the members of the groups may be attributed to the gain of accessory genes for their survival which in turn may be attributed to their variable genomic sizes, simple organization, or horizontal gene transfer rates (Kuo and Ochman, 2009; Aherfi et al., 2018). Also, the formation of ecotypes based on the varied isolation sources may not be feasible for groups of the genus *Roseomonas.* This is also evident from the distinctive genomic features observed during the pan-genome evaluation for each of the groups, which may have resulted in response to the environmental changes.

The extensive taxogenomics study performed substantiates the separation of the respective groups of the genus *Roseomonas* into separate genera as suggested by Romano-Bertrand et al. (2016) and Hördt et al. (2020). Considering the fact that *Roseomonas* is a very divergent genus, henceforth members of this genus cannot be clustered under one phylogenetic genus, necessitating the creation of six new genera. For each of the novel genera, emended description and reclassification are given. Phylogenetic trees based on the 16S rRNA gene (Figure 1) and whole genome sequences (Figure 2) agree with the formation of seven different clades as separate genera. Although the 16S rRNA gene studies have certain drawbacks, this approach is still quite accountable for the delineation of the members of the genus *Roseomonas*. This study would be a first attempt in re-evaluating the genus *Roseomonas* and reclassifying it by creating six new genera: Group II as *Pararoseomonas* gen. nov., Group III as *Falsiroseomonas* gen. nov., Group IV as *Paeniroseomonas* gen. nov., Group V as *Plastoroseomonas* gen. nov., Group VI as *Neoroseomonas* gen. nov., and Group VII as *Pseudoroseomonas* gen. nov. The genus delineation for Group IV is based on the 16S rRNA gene analysis (in the absence of genome sequences) and phenotypic characteristics. The genus delineation constructed from the 16S rRNA gene sequences and phenotypic characters provide sufficient resolution to distinguish the Group IV members from other groups of the genus *Roseomonas*. However, the availability of the genome sequences may further substantiate the reclassification in the future. The species within each of the newly defined genera have been defined accurately as per the recommended standards for ANI and *d*DDH (Supplementary Table 5). As for the genus delineation, the inter-group AAI and POCP values agree with the standards given for genus determination (Supplementary Tables 6, 7). The justification for genus delineation based on the 16S rRNA gene identity, AAI and POCP, and other phenotypic characters are given in Figure 4 and Table 2.

### Emended Description of the Genus *Roseomonas* by Rihs et al. (1998)

The description is the same as the one given by Rihs et al. (1993) and Sánchez-Porro et al. (2009) for the genus *Roseomonas* except for a few modifications. H_2_S formation is variable and the genome-based G+C content is ∼70 mol%.

The type species is *Roseomonas gilardii*.

### Description of *Pararoseomonas* gen. nov.

*Pararoseomonas* (Pa.ra.ro.se.o.mo’nas. Gr. pref. *para*-, next to, resembling; N.L. fem. n. *Roseomonas*, a bacterial genus; N.L. fem. n. *Pararoseomonas*, next to *Roseomonas*).

Members are aerobic, Gram-negative, coccoid or short rods, and non-motile. Variable in both oxidase and catalase. Polar lipids consist of diphosphatidylglycerol, phosphatidylglycerol, phosphatidylethanolamine, phosphatidylcholine, unidentified phospholipid, and unidentified aminolipids. Summed feature 3, C_18:1_ 2-OH, C_18:1_ 3-OH, C_16:0,_ C_18:0_ are the major fatty acids. Members have a genomic size of 4.9–6.4 Mbp and a genomic G+C content of 69.7–71.4 mol%. The genus delineation is based on the 16S rRNA gene, 92 core genes (phylogenomics), AAI indices, POCP values, and phenotypic and genomic features.

The type species is *Pararoseomonas rosea.*

### Description of *Pararoseomonas rosea* comb. nov.

*Pararoseomonas rosea* (ro’se.a. L. fem. adj. *rosea* rose-colored, pink).

#### Basonym: *Roseomonas rosea* (Kämpfer et al., 2003; Sánchez-Porro et al., 2009)

The description is the same as that given for *Roseomonas rosea* by Sánchez-Porro et al. (2009). AJ488505 is the accession number for the 16S rRNA gene sequence and FQZF00000000 for the genome sequence. The type strain is 173/96^T^ (=CIP 107419^T^ = DSM 14916^T^).

### Description of *Pararoseomonas vinacea* comb. nov.

*Pararoseomonas vinacea* (vi.na’ce.a. L. fem. adj. *vinacea*, of or belonging to wine or to the grape, referring to the colony color).

#### Basonym: *Roseomonas vinacea* (Zhang et al., 2008)

The description is same as that given for *Roseomonas vinacea* by Zhang et al. (2008). The accession numbers for the 16S rRNA gene and genome sequences are MT760143 and BO93DRAFT, respectively. The type strain is CPCC 100056^T^ (=KCTC 22045^T^ = CCM 7468^T^).

### Description of *Pararoseomonas nepalensis* comb. nov.

*Pararoseomonas nepalensis* (ne.pal.en’sis. N.L. fem. adj. *nepalensis*, pertaining to Nepal, the country where source soil samples were collected).

#### Basonym: *Roseomonas nepalensis* (Chaudhary and Kim, 2017)

The description is the same as the one given for *Roseomonas nepalensis* by Chaudhary and Kim (2017). The accession number for the 16S rRNA gene sequence is KX129819. The type strain is G-3-5^T^ (=JCM 31470^T^ = KACC 18908^T^).

### Description of *Pararoseomonas aeriglobus* comb. nov.

*Pararoseomonas aeriglobus* (a.e.ri.glo’bus Gr. masc. n. *aêr*, air; L. masc. n. *globus*, a sphere; N.L. masc. n. *aeriglobus*, a sphere living in air).

#### Basonym: *Roseomonas aeriglobus* (Lee and Jeon, 2018)

The description of *Pararoseomonas aeriglobus* is the same as that of *Roseomonas aeriglobus* as given by Lee and Jeon (2018). The accession number for the 16S rRNA gene sequence is KY864922. The type strain is KER25-12^T^ (=KACC 19282^T^ = JCM 32049^T^).

### Description of *Pararoseomonas aerilata* comb. nov.

*Pararoseomonas aerilata* (ae.ri.la’ta. Lat. masc. n. *aêr*, air; L. fem. perf. part. *lata*, carried; N.L. fem. part. adj. *aerilata*, airborne).

#### Basonym: *Roseomonas aerilata* (Yoo et al., 2008)

The description is the same as that of *Roseomonas aerilata* as given by Yoo et al. (2008). Accession numbers of the 16S rRNA gene and genome sequences are EF661571 and JONP00000000, respectively. The type strain is 5420S-30^T^ (=KACC 12521^T^ = DSM 19363^T^).

### Description of *Pararoseomonas radiodurans* comb. nov.

*Pararoseomonas radiodurans* (ra.di.o.du’rans. L. masc. n. *radius*, a beam or ray; N.L. pref. *radio*-, pertaining to radiation; L. pres. part. *durans*, enduring; N.L. part. adj. *radiodurans*, resisting radiation).

#### Basonym: *Roseomonas radiodurans* (Kim et al., 2018)

The description is the same as that given by Kim et al. (2018) for *Roseomonas radiodurans*. The accession number for the 16S rRNA gene sequence is KY887689.

The type strain is 17Sr1-1^T^ (=KCTC 52899^T^ = NBRC 112872^T^).

### Description of *Pararoseomonas pecuniae* comb. nov.

*Pararoseomonas pecuniae* (pe.cu’ni.ae. L. gen. n. *pecuniae*, of/from money or a coin, referring to the source of isolation of the type strain).

#### Basonym: *Roseomonas pecuniae* (Lopes et al., 2011)

The description given is the same as that of *Roseomonas pecuniae* as given by Lopes et al. (2011). GU168019 is the accession number for the 16S rRNA gene sequence. The accession number for the genome sequence is JACIJD00000000. The type strain is N75^T^ (=LMG 25481^T^ = CIP 110074^T^).

### Description of *Pararoseomonas harenae* comb. nov.

*Pararoseomonas harenae* (ha.re’nae. L. gen. fem. n. *harenae*, of sand, referring to the isolation of the type strain from desert sand).

#### Basonym: *Roseomonas harenae* (Deng et al., 2020)

The description is the same as that of *Roseomonas harenae* as given by Deng et al. (2020). MN883396 and WWDL00000000 are the accession numbers for the 16S rRNA gene and genome sequences, respectively. The type strain is CPCC 101081^T^ (=KCTC 62852^T^ = NBRC 113512^T^).

### Description of *Falsiroseomonas* gen. nov.

*Falsiroseomonas* (Fal’si.ro.se.o.mo’nas. L. adj. *falsus*, false; N.L. fem. n. *Roseomonas*, a bacterial genus name; N.L. fem. n. *Falsiroseomonas*, false *Roseomonas*).

Members of this genus are aerobic, Gram-negative, and coccoid-rod in shape. Motility varies within the genus. Oxidase positive and catalase variable. Polar lipids consist of diphosphatidylglycerol, phosphatidylglycerol, phosphatidylethanolamine, phosphatidylcholine, unidentified phospholipid, unidentified glycolipid, and unidentified aminolipid. Summed feature 3, C_16:1_ *ω*_5c__ and_ C_16:0_ are the major fatty acids. Members have a genomic size of 5.8–6.6 Mb and a genomic G+C content of 70–72 mol%. The genus delineation is based on the 16S rRNA gene, 92 core genes (phylogenomics), AAI, POCP values, and phenotypic and genomic features.

The type species is *Falsiroseomonas stagni*.

### Description of *Falsiroseomonas stagni* comb. nov.

*Falsiroseomonas stagni* (stag’ni. L. gen. neut. n. *stagni*, of a pond, indicating the site of isolation of this organism).

#### Basonym: *Roseomonas stagni* (Furuhata et al., 2008)

The species description is the same as that of *Roseomonas stagni* as given by Furuhata et al. (2008). AB369258 is the accession number for the 16S rRNA gene sequence and FOSQ00000000 for the genome sequence. The type strain is HS-69^T^ (=DSM 19981^T^ = JCM 15034^T^ = KCTC 22213^T^).

### Description of *Falsiroseomonas bella* comb. nov.

*Falsiroseomonas bella* (bel’la. L. fem. adj. *bella*, pretty).

#### Basonym: *Roseomonas bella* (Zhang et al., 2020)

The description is the same as that given by Zhang et al. (2020) for *Roseomonas bella*. MG601506 is the accession number for the 16S rRNA gene sequence and QGNA00000000 for the genome sequence. The type strain is CQN31^T^ (=KCTC 62447^T^ = MCCC 1H00309^T^).

### Description of *Falsiroseomonas wooponensis* comb. nov.

*Falsiroseomonas wooponensis* (woo.po.nen’sis. N.L. masc./fem. adj. *wooponensis*, of or belonging to Woopo wetland, South Korea, the geographical origin of the type strain of the species).

#### Basonym: *Roseomonas wooponensis* (Lee et al., 2015)

The description is the same as that of *Roseomonas wooponensis* as given by Lee et al. (2015). KF619243 is the accession number for the 16S rRNA gene sequence. The type strain is WW53^T^ (=KCTC 32534^T^ = JCM 19527^T^).

### Description of *Falsiroseomonas terricola* comb. nov.

*Falsiroseomonas terricola* (ter.ri’co.la. L. fem. n. *terra*, earth, soil; L. masc./fem. suff. -*cola*, inhabitant, dweller; from L. masc./fem. n. *incola*, dweller; N.L. masc./fem. n. *terricola*, a dweller upon earth, soil-dweller, referring to the isolation of the type strain from soil).

#### Basonym: *Roseomonas terricola* (Kim et al., 2017)

The description is the same as that given for *Roseomonas terricola* by Kim et al. (2017). FJ654263 is the accession number for the 16S rRNA gene sequence. The type strain is EM302^T^ (=KACC 13942^T^ = KCTC 42906^T^ = NBRC 111477^T^).

### Description of *Falsiroseomonas selenitidurans* comb. nov.

*Falsiroseomonas selenitidurans* (se.le.ni.ti.du’rans. N.L. neut. n. *selenitum*, selenite; L. v. *duro*, withstand; N.L. part. adj. *selenitidurans*, withstanding selenite).

#### Basonym: *Roseomonas selenitidurans* (Hou et al., 2020)

The description is the same as that given for *Roseomonas selenitidurans* by Hou et al. (2020). MT107132 is the accession number for the 16S rRNA gene sequence and JAAVNE00000000 for the genome sequence. The type strain is BU-1^T^ (=GDMCC 1.1776^T^ = KACC 21750^ T^).

### Description of *Falsiroseomonas frigidaquae* comb. nov.

*Falsiroseomonas frigidaquae* (fri.gi.da’quae. L. masc. adj. *frigidus*, cold; L. fem. n. *aqua*, water; N.L. gen. fem. n. *frigidaquae*, from/of cold water, referring to the isolation of the type strain from a water-cooling system).

#### Basonym: *Roseomonas frigidaquae* (Kim et al., 2009)

The description is the same as that given by Kim et al. (2009) for *Roseomonas frigidaquae.* EU210160 is the accession number for the 16S rRNA gene sequence and JAAVTX00000000 for the genome sequence. The type strain is CW67^T^ (=JCM 15073^T^ = KCTC 22211^T^).

### Description of *Falsiroseomonas tokyonensis* comb. nov.

*Falsiroseomonas tokyonensis* (to.ky.o.nen’sis. N.L. masc./fem. adj. *tokyonensis*, of Tokyo, from where the strain was isolated).

#### Basonym: *Roseomonas tokyonensis* (Furuhata et al., 2014)

The description is the same as the one given for *Roseomonas tokyonensis* by Furuhata et al. (2013). AB297501 is the gene accession number for the 16S rRNA gene sequence. The type strain is K-20^T^ (=JCM 14634^T^ = KCTC 32152^T^).

### Description of *Falsiroseomonas riguiloci* comb. nov.

*Falsiroseomonas riguiloci* (ri.gu.i.lo’ci. L. masc. adj. *riguus*, well-watered; L. masc. n. *locus*, a site; N.L. gen. n. *riguiloci*, from a well-watered place where the type strain was isolated).

#### Basonym: *Roseomonas riguiloci* (Baik et al., 2012)

The description is the same as that given by Baik et al. (2012) for *Roseomonas riguiloci.* HQ436503 is the accession number for the 16S rRNA gene sequence. The type strain is 03SU10-P^T^ (=KCTC 23339^T^ = JCM 17520^T^ = DSM 29515^T^).

### Description of *Falsiroseomonas algicola* comb. nov.

*Falsiroseomonas algicola* (al.gi’co.la. L. fem. n. *alga* (*gen. algae*), an alga; L. masc./fem. suff. -*cola*, dweller; from L. masc./fem. n. *incola*, an inhabitant; N.L. masc./fem. n. *algicola*, an inhabitant of algae).

#### Basonym: *Roseomonas algicola* (Kim et al., 2020)

The description is the same as the one given by Kim et al. (2020) for *Roseomonas algicola.* MK342491 is the accession number for the 16S rRNA gene sequence and JAAIKB00000000 for the genome sequence. The type strain is PeD5^T^ (=JCM 33309^T^ = KACC 19925^T^).

### Description of *Falsiroseomonas arcticisoli* com. nov.

***Falsiroseomonas arcticisoli*** (arc’ti.cus arc.ti.ci.so’li L. masc. adj. *arcticus*, northern; L. neut. n. *solum*, soil; N.L. gen. n. *arcticisoli*, of soil from the Arctic).

#### Basonym: *Roseomonas arcticisoli* (Kim M. C. et al., 2016)

The description is the same as the one given by Kim M. C. et al. (2016) for *Roseomonas arcticisoli.* KP274055 is the accession number for the 16S rRNA gene sequence. The type strain is MC 3624^T^ (=CCTCC AB 2014278^T^ = LMG 28637^T^).

### Description of *Paeniroseomonas* gen. nov.

*Paeniroseomonas* (Pa.e.ni.ro.se.o.mo’nas. L. adv. *paene*, almost; - i-, connecting vowel; N.L. fem. n. *Roseomonas*, a bacterial genus; N.L. fem. n. *Paeniroseomonas*, almost a *Roseomonas*).

Members of this genus are strictly aerobic, Gram-negative, and coccoid-rod shaped. Cells variable motile. Oxidase negative and catalase positive. Diphosphatidylglycerol, phosphatidylglycerol, phosphatidylethanolamine, and phosphatidylcholine are the major polar lipids. Summed feature 3, C_18:1_ *ω*7c, C_18:1_ 2-OH, and C_16:0_ are the major fatty acids. G+C% content of 68.6–73.1 (mol%). The genus delineation is based on the 16S rRNA gene-based phylogeny and phenotypic features.

The type species is *Paeniroseomonas aquatica.*

### Description of *Paeniroseomonas aquatica* comb. nov.

*Paeniroseomonas aquatica* (a.qua’ti.ca L. fem. adj. *aquatica*, found in water, aquatic).

#### Basonym: *Roseomonas aquatica* (Gallego et al., 2006)

The description is the same as that given by Gallego et al. (2006) for *Roseomonas aquatica.* AM231587 is the accession number for the 16S rRNA gene sequence. The type strain is TR53^T^ (=CECT 7131^T^ = JCM 13556^T^ = DSM 19438^T^).

### Description of *Paeniroseomonas fluminis* comb. nov.

*Paeniroseomonas fluminis* (flu’mi.nis L. gen. neut. n. *fluminis*, of a river).

#### Basonym: *Roseomonas fluminis* (Ko et al., 2018)

The description is the same as that of *Roseomonas fluminis* as given by Ko et al. (2018). KY649439 is the accession number for the 16S rRNA gene sequence. The type strain is D3^T^ (=JCM 31968^T^ = KACC 19269^T^).

### Description of *Plastoroseomonas* gen. nov.

*Plastoroseomonas* (Plas.to.ros.e.o.mo’nas. Gr. adj. *plastos*, false; N.L. fem. n. *Roseomonas*, a bacterial genus; N.L. fem. n. *Plastoroseomonas*, a false *Roseomonas*).

Members of the genus are aerobic, Gram-negative, and rod shaped. Cells are motile. Oxidase negative and catalase positive. Diphosphatidylglycerol, phosphatidylglycerol, phosphatidylethanolamine, unidentified aminolipid, and unidentified lipid are the major polar lipids. Summed feature 3, C_18:1_ 2-OH and C_16:0_ are the major fatty acids. Genomic size of members ranges from 4.4 to 7.2 Mb and G+C content is ∼70 mol%. The genus delineation is based on the 16S rRNA gene, 92 core genes (phylogenomic), AAI indices, POCP values, and genomic and phenotypic features.

The type species is *Plastoroseomonas arctica.*

### Description of *Plastoroseomonas arctica* comb. nov.

*Plastoroseomonas arctica* (arc’ti.ca. L. fem. adj. *arctica*, northern, from the Arctic, referring to the site where the type strain was isolated).

#### Basonym: *Roseomonas arctica* (Qiu et al., 2016)

The description is the same as that of *Roseomonas arctica* as given by Qiu et al. (2016). KJ647399 and JAAEDH000000000 are the accession numbers for the 16S rRNA gene and genome sequences, respectively. The type strain is M6-79^T^ (=CCTCC AB 2013101^T^ = LMG 28251^T^).

### Description of *Plastoroseomonas hellenica* comb. nov.

*“Plastoroseomonas hellenica”* (hel-lé-ni-ka. Gr. adj. *ellenikos*, Greek, L. fem. adj. *hellenica*, Greek, pertaining to Greece, the country from where the bacterium was first isolated).

#### Basonym: *Roseomonas hellenica* (Rat et al., 2021)

The description is the same as that of *Roseomonas hellenica* as given by Rat et al. (2021). MN647549 and JAAGBB000000000 are the accession numbers for the 16S rRNA gene and genome sequences, respectively. The type strain is R-73080^T^ (=LMG 31523^T^ = CECT 30032^T^).

### Description of *Pseudoroseomonas* gen. nov.

*Pseudoroseomonas* (Pseu.do.ro.se.o.mo’nas. Gr. adj. *pseudês*, false; N.L. fem. n. *Roseomonas*, a bacterial genus; N.L. fem. n. *Pseudoroseomonas*, false *Roseomonas*).

Members of the genus are aerobic, Gram-negative, and coccoid to short rods in shape. Cells are non-motile. Oxidase positive and catalase positive. Diphosphatidylglycerol, phosphatidylglycerol, phosphatidylethanolamine, unidentified aminolipid, unidentified lipid, and unidentified phospholipid are the major polar lipids. Summed feature 3, C_18:1_ 2-OH and C_16:0_ are the major fatty acids. Genomic size of members ranges from 4.2 to 6.4 Mb and G+C% content is 68.7–72.7%. The genus delineation is based on the 16S rRNA gene, 92 core genes (phylogenomic), AAI indices, POCP values, and phenotypic and genomic features.

The type species is *Pseudoroseomonas cervicalis.*

### Description of *Pseudoroseomonas cervicalis* comb. nov.

*Pseudoroseomonas cervicalis* [cer.vi.ca’lis. L. fem. n. *cervix (gen. cervicis)*, neck; L. masc./fem. Adj. suff. -*alis*, suffix denoting pertaining to; N.L. fem. adj. *cervicalis*, pertaining to cervix, from the cervix].

#### Basonym: *Roseomonas cervicalis* (Rihs et al., 1998)

The description is the same as that of *Roseomonas cervicalis* as given by Rihs et al. (1993). AF533353 is the accession number for the 16S rRNA gene sequence and ADVL00000000 for the genome sequence. The type strain is E7107^T^ (=ATCC 49957^T^ = CIP 104027^T^).

### Description of *Pseudoroseomonas suffusca* comb. nov.

*Pseudoroseomonas suffusca* (suf.fus’ca. L. fem. adj. *suffusca*, light brown, referring to the color of colonies).

#### Basonym: *Roseomonas suffusca* (Subhash and Lee, 2017)

The description is the same as that of *Roseomonas suffusca* as given by Subhash and Lee (2017). LT009497 is the accession number for the 16S rRNA gene sequence. The type strain is S1^T^ (=KEMB 563-465^T^ = JCM 31176^T^).

### Description of *Pseudoroseomonas rubra* comb. nov.

*Pseudoroseomonas rubra* (ru’bra. L. fem. adj. *rubra*, red).

#### Basonym: *Roseomonas rubra* (Subhash et al., 2016)

The description is the same as that of *Roseomonas rubra* as given by Subhash et al. (2016). LT009499 is the accession number for the 16S rRNA gene sequence. The type strain is S5^T^ (=JCM 31177^T^ = KEMB 563-468^T^).

### Description of *Pseudoroseomonas hibiscisoli* comb. nov.

*Pseudoroseomonas hibiscisoli* (hi.bis.ci.so’li. N.L. masc. n. *Hibiscus*, Mugunghwa/Hibiscus syriacus; L. neut. n. *solum*, soil; N.L. gen. neut. n. *hibiscisoli*, of soil of a *Hibiscus*, the source of the type strain).

#### Basonym: *Roseomonas hibiscisoli* (Yan et al., 2017)

The description is the same as that of *Roseomonas hibiscisoli* as given by Yan et al. (2017). KX456186 is the accession number for the 16S rRNA gene sequence. The type strain is THG-N2.22^T^ (=KACC 18935^T^ = CCTCC AB 2016176^T^).

### Description of *Pseudoroseomonas rhizosphaerae* comb. nov.

*Pseudoroseomonas rhizosphaerae* (rhi.zo.sphae’rae. Gr. fem. n. *rhiza*, a root; Gr. fem. n. *sphaîra*, a ball, a sphere; N.L. fem. n. *rhizosphaera*, the rhizosphere; N.L. gen. fem. N. *rhizosphaerae*, of the rhizosphere).

#### Basonym: *Roseomonas rhizosphaerae* (Chen et al., 2014)

The description is the same as that of *Roseomonas rhizosphaerae* as given by Chen et al. (2014). KC904962 is the accession number for the 16S rRNA gene sequence and PDNU00000000 for the genome sequence. The type strain is YW11^T^ (= KACC 17225^T^ = CCTCC AB2013041^T^).

### Description of *Pseudoroseomonas aestuarii* comb. nov.

*Pseudoroseomonas aestuarii* (aes.tu.a’ri.i. L. gen. n. *aestuarii*, of an estuary, the habitat from which the type strain was isolated).

#### Basonym: *Roseomonas aestuarii* (Venkata Ramana et al., 2010)

The description is the same as that of *Roseomonas aestuarii* as given by Venkata Ramana et al. (2010). AB682256 is the accession number for the 16S rRNA gene sequence. The type strain is JC17^T^ (=CCUG 57456^T^ = KCTC 22692^T^ = NBRC 105654^T^).

### Description of *Pseudoroseomonas aerofrigidensis* comb. nov.

*Pseudoroseomonas aerofrigidensis* (a.e.ro.fri.gi.den’sis. Gr. masc. n. *aêr*, air; L. masc. adj. *frigidus*, cold, cool, chilled; L. masc./fem. adj. suff. -*ensis*, suffixes used in the sense of “belonging to” or “coming from”; N.L. fem. adj. *aerofrigidensis*, pertaining to cooling air, as the strain was isolated from an air conditioner).

#### Basonym: *Roseomonas aerofrigidensis* (Hyeon and Jeon, 2017)

The description is the same as that of *Roseomonas aerofrigidensis* as given by Hyeon and Jeon (2017). KY126356 is the accession number for the 16S rRNA gene sequence. The type strain is HC1^T^ (=JCM 31878^T^ = KACC 19097^T^).

### Description of *Pseudoroseomonas oryzae* comb. nov.

*Pseudoroseomonas oryzae* (o.ry’zae. L. gen. fem. n. *oryzae*, of rice, pertaining to the isolation of the type strain from rice paddy soil).

#### Basonym: *Roseomonas oryzae* (Ramaprasad et al., 2015)

The description is to the same as that of *Roseomonas oryzae*, as described by Ramaprasad et al. (2015). LN810637 is the accession number for the 16S rRNA gene sequence and VUKA00000000 for the genome sequence. The type strain is JC288^T^ (=KCTC 42542^T^ = LMG 28711^T^).

### Description of *Pseudoroseomonas vastitatis* comb. nov.

*Pseudoroseomonas vastitatis* (vas.ti.ta’tis. L. gen. fem. n. *vastitatis*, of a desert, referring to the isolation source of the type strain).

#### Basonym: *Roseomonas vastitatis* (Zhao et al., 2020)

The description is the same as that of *Roseomonas vastitatis* as given by Zhao et al. (2020). MK421542 is the accession number for the 16S rRNA gene sequence and QXGS00000000 for the genome sequence. The type strain is CPCC 101021^T^ (=J1A743^T^ = KCTC 62043^T^).

### Description of *Pseudoroseomonas globiformis* comb. nov.

*Pseudoroseomonas globiformis* (glo.bi.for’mis. L. masc. n. *globus*, sphere; L. fem. n. *forma*, shape; N.L. masc./fem. adj. *globiformis*, of spherical shape).

#### Basonym: *Roseomonas globiformis* (Fang et al., 2018)

The description is to the same as that of *Roseomonas globiformis* as given by Fang et al. (2018). MG589944 is the accession number for the 16S rRNA gene sequence. The type strain is CPCC 100847^T^ (=KCTC 52094^T^).

### Description of *Pseudoroseomonas wenyumeiae* comb. nov.

*Pseudoroseomonas wenyumeiae* (wen.yu.mei’ae. N.L. gen. fem. n. *wenyumeiae*, of Yumei Wen, a famous microbiologist, for her contribution to the Hepatitis B vaccine and anti-HBs complex research and her fundamental role in immunology in China).

#### Basonym: *Roseomonas wenyumeiae* (Tian et al., 2019)

The description is the same as that of *Roseomonas wenyumeiae* as given by Tian et al. (2019). MH974806 is the accession number for the 16S rRNA gene sequence and RFLX00000000 for the genome sequence. The type strain is Z23^T^ (=CGMCC 1.16540^T^ = DSM 106207^T^).

### Description of *Pseudoroseomonas ludipueritiae* comb. nov.

*Pseudoroseomonas ludipueritiae* [lu.di.pu.e.ri’ti.ae. L. masc. n. *ludus*, a place of exercise or practice, a school for elementary instruction; L. masc. n. *puer (gen. pueri)*, a child; N.L. gen. n. *ludipueritiae*, of a playing place of childhood, intended to mean a kindergarten].

#### Basonym: *Roseomonas ludipueritiae* (Kämpfer et al., 2003; Sánchez-Porro et al., 2009)

The description is the same as that of *Roseomonas ludipueritia* as given by Kämpfer et al. (2003) and Sánchez-Porro et al. (2009). AJ488504 is the accession number for the 16S rRNA gene sequence and JACTUZ0000000 for the genome sequence. The type strain is 170-96^T^ (=CIP 107418^T^ = DSM 14915^T^).

### Description of *Pseudoroseomonas aerophila* comb. nov.

*Pseudoroseomonas aerophila* (a.e.ro’phi.la. Gr. masc. n. *aêr*, air; Gr. masc. adj. *philos*, loving; N.L. fem. adj. *aerophila*, air-loving).

#### Basonym: *Roseomonas aerophila* (Kim et al., 2013)

The description is the same as that of *Roseomonas aerophile* as given by Kim et al. (2013). JX275860 is the accession number for the 16S rRNA gene sequence and JACTVA00000000 for the genome sequence. The type strain is 7515T-07^T^ (=KACC 16529^T^ = NBRC 108923^T^).

### Description of *Pseudoroseomonas musae* comb. nov.

*Pseudoroseomonas musae* (mu’sae. L. gen. fem. n. *musae*, of Musa, isolated from leaf of banana *Musa* sp).

#### Basonym: *Roseomonas musae* (Nutaratat et al., 2017)

The description is the same as that of *Roseomonas musae* as given by Nutaratat et al. (2013). AB594201 is the accession number for the 16S rRNA gene sequence. The type strain is PN1^T^ (=BCC 44863^T^ = NBRC 107870^T^).

### Description of *Pseudoroseomonas coralli* comb. nov.

*Pseudoroseomonas coralli* (co.ral’li. L. gen. n. *coralli*, of coral, from which the organism was isolated).

#### Basonym: *Roseomonas coralli* (Li et al., 2021)

The description is the same as that of *Roseomonas coralli* as given by Li et al. (2021). MN336179 is the accession number for the 16S rRNA gene sequence and SNVJ000000000 for the genome sequence. The type strain is M0104^T^ (=KCTC 62359^T^ = MCCC 1K03632^T^).

### Description of *Pseudoroseomonas deserti* comb. nov.

*Pseudoroseomonas deserti* (de.ser’ti. L. gen. neut. n. *deserti*, of a desert).

#### Basonym: *Roseomonas deserti* (Subhash and Lee, 2018)

The description is the same as that of *Roseomonas deserti* as given by Subhash and Lee (2018). LT837512 is the accession number for the 16S rRNA gene sequence and MLCO00000000 for the genome sequence. The type strain is M3^T^ (=KEMB 2255-459^T^ = JCM 31275^T^).

### Description of *Neoroseomonas* gen. nov.

*Neoroseomonas* (Ne.o.ro.se.o.mo’nas. Gr. adj. *neos*, new; N.L. fem. n. *Roseomonas*, a bacterial genus; N.L. fem. n. *Neoroseomonas*, to refer to the fact that it is a new group of *Roseomonas*).

Members are aerobic, Gram-negative, and coccoid to short rod in shape. Motility is variable within the members. Oxidase and catalase positive. Diphosphatidylglycerol, phosphatidylglycerol, phosphatidylcholine, phosphatidylethanolamine, unidentified glycolipid, unidentified aminolipid, unidentified lipid, and unidentified phospholipid are the major polar lipids. Summed feature 3, C_18:1_ 2-OH and C_16:0_ are the major fatty acids. Genome size of members ranges from 4.7 to 6.3 Mb and G+C% content is 68.8–71.5%. The genus delineation is based on the 16S rRNA gene, 92 core genes (phylogenomic), AAI indices, POCP values, and phenotypic and genomic features.

The type species is *Neoroseomonas lacus.*

### Description of *Neoroseomonas lacus* comb. nov.

*Neoroseomonas lacus* (la’cus. L. gen. masc. n. *lacus*, of a lake, indicating the site of isolation of this organism).

#### Basonym: *Roseomonas lacus* (Jiang et al., 2006)

The description is the same as that of *Roseomonas lacu* as given by Jiang et al. (2006). AJ786000 is the accession number for the 16S rRNA gene sequence and BMKW00000000 for the genome sequence. The type strain is TH-G33^T^ (=CGMCC 1.3617^T^ = JCM 13283^T^).

### Description of *Neoroseomonas terrae* comb. nov.

*Neoroseomonas terrae* (ter’rae. L. gen. fem. n. *terrae*, of the soil).

#### Basonym: *Roseomonas terrae* (Yoon et al., 2007)

The description is the same as that of *Roseomonas terrae* as given by Yoon et al. (2007). EF363716 and JAAEDI000000000 are the accession numbers for the 16S rRNA gene and genome sequences, respectively. The type strain is DS-48^T^ (=KCTC 12874^T^ = JCM 14592^T^).

### Description of *Neoroseomonas eburnea* comb. nov.

*Neoroseomonas eburnea* (e.bur’ne.a. L. fem. adj. *eburnea*, white as ivory).

#### Basonym: *Roseomonas eburnea* (Wang et al., 2016)

The description is the same as that of *Roseomonas eburnean* as given by Wang et al. (2016). KF254767 and JAAEDL000000000 are the accession numbers for the 16S rRNA gene and genome sequences, respectively. The type strain is BUT-5^T^ (=CCTCC AB2013276^T^ = KACC 17166^T^).

### Description of *Neoroseomonas alkaliterrae* comb. nov.

*Neoroseomonas alkaliterrae* (al.ka.li.ter’rae. Arabic masc. n. *al-qaliy*, the ashes of saltwort; N.L. neut. N. alkali, alkali; L. gen. fem. n. *terrae*, of the soil or earth; N.L. gen. fem. n. *alkaliterrae*, of alkaline soil).

#### Basonym: *Roseomonas alkaliterrae* (Dong et al., 2014)

The description is the same as that of *Roseomonas alkaliterrae* as given by Dong et al. (2014). KF771274 is the accession number for the 16S rRNA gene sequence and JACIJE00000000 for the genome sequence. The type strain is YIM 78007^T^ (=BCRC 80644^T^ = JCM 19656^T^ = DSM 25895^T^).

### Description of *Neoroseomonas oryzicola* comb. nov.

*Neoroseomonas oryzicola* (o.ry.zi’co.la. L. fem. n. *oryza*, rice; L. masc./fem. suff. -*cola*, an inhabitant; from L. masc./fem. n. *incola*, dweller; N.L. masc./fem. n. *oryzicola*, an inhabitant of rice).

#### Basonym: *Roseomonas oryzicola* (Chung et al., 2015)

The description is the same as that of *Roseomonas oryzicola* as given by Chung et al. (2015). EU707562 is the accession number for the 16S rRNA gene sequence and JAAVUP00000000 for the genome sequence. The type strain is YC6724^T^ (=KCTC 22478^T^ = NBRC 109439^T^).

### Description of *Neoroseomonas soli* comb. nov.

*Neoroseomonas soli* (so’li. L. gen. neut. n. *soli*, of soil, the source of the type strain).

#### Basonym: *Roseomonas soli* (Kim and Ka, 2014)

The description is the same as that of *Roseomonas soli* as given by Kim and Ka (2014). JN575264 and JAAEDM00000000 are the accession numbers for the 16S rRNA gene and genome sequences, respectively. The type strain is 5N26^T^ (=KACC 16376^T^ = NBRC 109097^T^).

### Description of *Neoroseomonas sediminicola* comb. nov.

*Neoroseomonas sediminicola* (se.di.mi.ni.co’la. L. neut. n. *sedimen -inis*, sediment; L. masc./fem. suff. -cola, inhabitant, dweller; N.L. masc./fem. n. *sediminicola*, sediment-dweller, referring to the source of the type strain).

#### Basonym: *Roseomonas sediminicola* (He et al., 2019)

The description is the same as that of *Roseomonas sediminicola* as given by He et al. (2014). JQ349047 is the accession number for the 16S rRNA gene sequence. The type strain is FW-3^T^ (=KACC 16616^T^ = JCM 18210^T^).

## Data Availability Statement

The datasets presented in this study can be found in online repositories. The names of the repository/repositories and accession number(s) can be found in the article/Supplementary Material.

## Author Contributions

AR, UJ, and NS designed the studies under the supervision of CS and CR. UJ, AR, NS, and GD performed the genomic and phylogenetic analysis. AR and UJ wrote the manuscript. CR and CS supervised the study, contributed to the text preparation, and revised the manuscript. All authors read and approved the final version of the manuscript.

## Conflict of Interest

The authors declare that the research was conducted in the absence of any commercial or financial relationships that could be construed as a potential conflict of interest.

## Publisher’s Note

All claims expressed in this article are solely those of the authors and do not necessarily represent those of their affiliated organizations, or those of the publisher, the editors and the reviewers. Any product that may be evaluated in this article, or claim that may be made by its manufacturer, is not guaranteed or endorsed by the publisher.

## Footnotes

1
https://lpsn.dsmz.de/family/acetobacteraceae

2
https://lpsn.dsmz.de/genus/roseomonas

3
http://ggdc.dsmz.de/ggdc.php#

4
http://enve-omics.ce.gatech.edu/aai/

5
https://software.broadinstitute.org/morpheus/

6
www.kbase.us

7
https://cge.cbs.dtu.dk/services/VirulenceFinder/

8
https://www.ezbiocloud.net/tools/pairAlign

## Abbreviations

NCBI: National Center for Biotechnology Information
ANI: Average Nucleotide Identity
AAI: Average Amino acid Identity
POCP: Percentage of Conserved Proteins
*d*DDH: digital DNA-DNA Hybridization
BLAST: Basic Local Alignment Search Tool
MUSCLE: MUltiple Sequence Comparison by Log-Expectation
KCTC: Korean Collection for Type Cultures
CGMCC: China General Microbiological Culture Collection Centre
ATCC: American Type Culture Collection
CIP: Institute Pasteur Collection
DSM: Deutsche Sammlung von Mikroorganismen und Zellkulturen GmbH
JCM: Japan Collection of Microorganisms-RIKEN BioResource Center
KACC: Korean Agricultural Culture Collection
NBRC: NITE Biological Resource Center
LMG: Laboratorium voor Microbiologie
MCCC: Marine Culture Collection of China
GDMCC: Guangdong Microbial Culture Collection Center
CECT: Colección Española de Cultivos Tipo
KEMB: Korea Environmental Microorganisms Bank
CCUG: Culture Collection University of Göteborg
BCC: BIOTEC Culture Collection
UBCG: Up-to-date Bacterial Core Gene set.
