# Supplementary material for: Phylotaxogenomics for the Reappraisal of the Genus Roseomonas With the Creation of Six New Genera
Source: Front Microbiol. 2021 Aug 13;12:677842. doi: 10.3389/fmicb.2021.677842 (PMC8414978; doi:10.3389/fmicb.2021.677842)
Supplement: Supplementary file 1 [file Data_Sheet_1.PDF]

Taxogenomics for the reappraisal of the genus *Roseomonas* with the creation of six new genera

Anusha Rai[1#]., Uppada Jagadeshwari[2,#], Gupta **Deepshikha[1]., Nandardhane Smita[1].,**
[2*]Chintalapati Sasikala and [1*]Chintalapati Venkata Ramana

[1]Department of Plant Sciences, School of Life Sciences, University of Hyderabad, P.O. Central University,
Hyderabad 500046, INDIA

[2]Bacterial Discovery Laboratory, Centre for Environment, Institute of Science and Technology, J. N. T. University, Hyderabad, Kukatpally, Hyderabad-500085. INDIA

*Authors for correspondence: Ramana, Ch. V., Sasikala, Ch.
E-mail: cvr449@gmail.com; sasi449@yahoo.ie; sasikala.ch@gmail.com

Short title: Reclassification of the genus *Roseomonas*

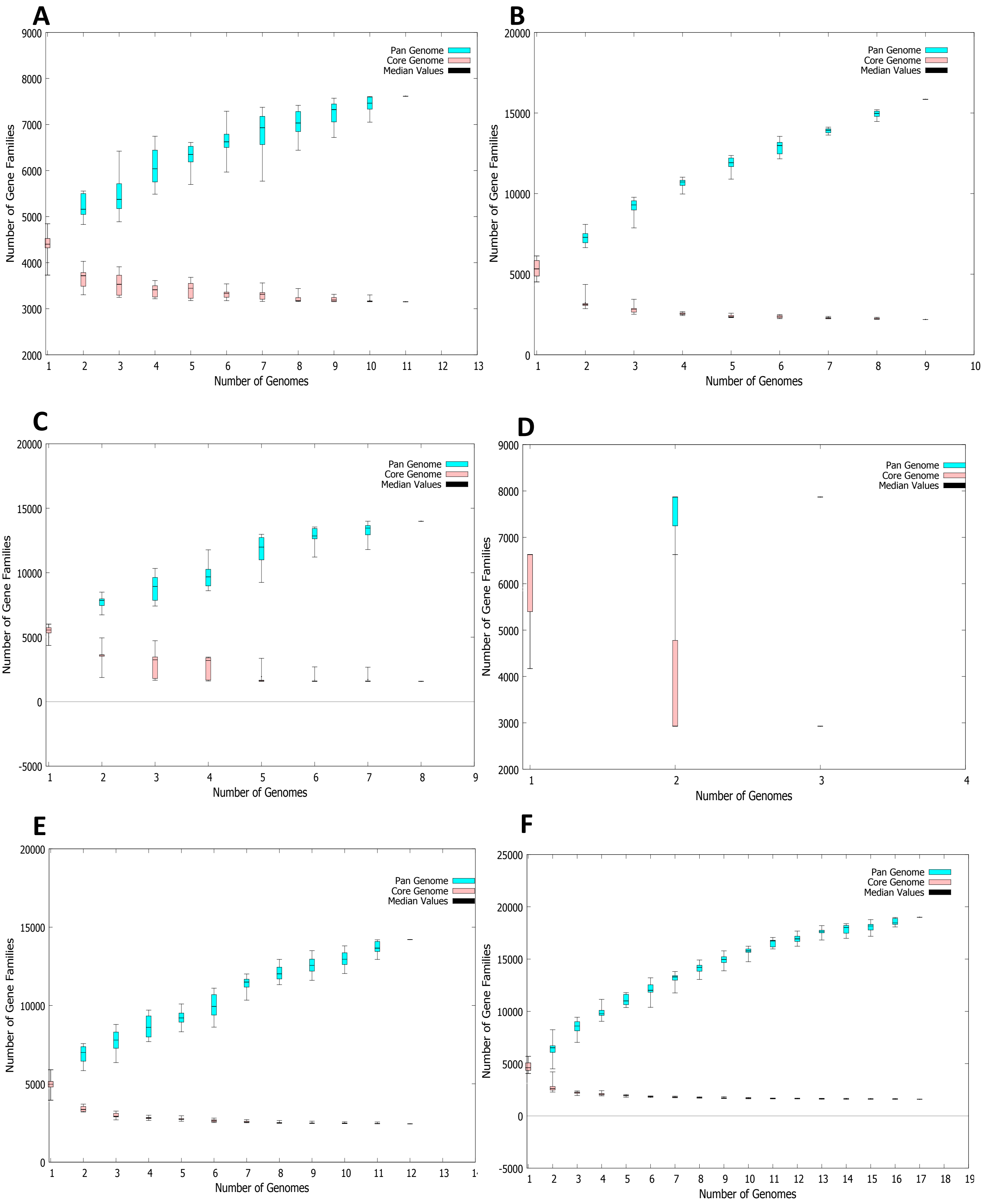

Supplementary Figure S1. Core and pan-genome plot of members of (A) Group I (B) Group II (C) Group III (D) Group V (E) Group VI (F) Group VII

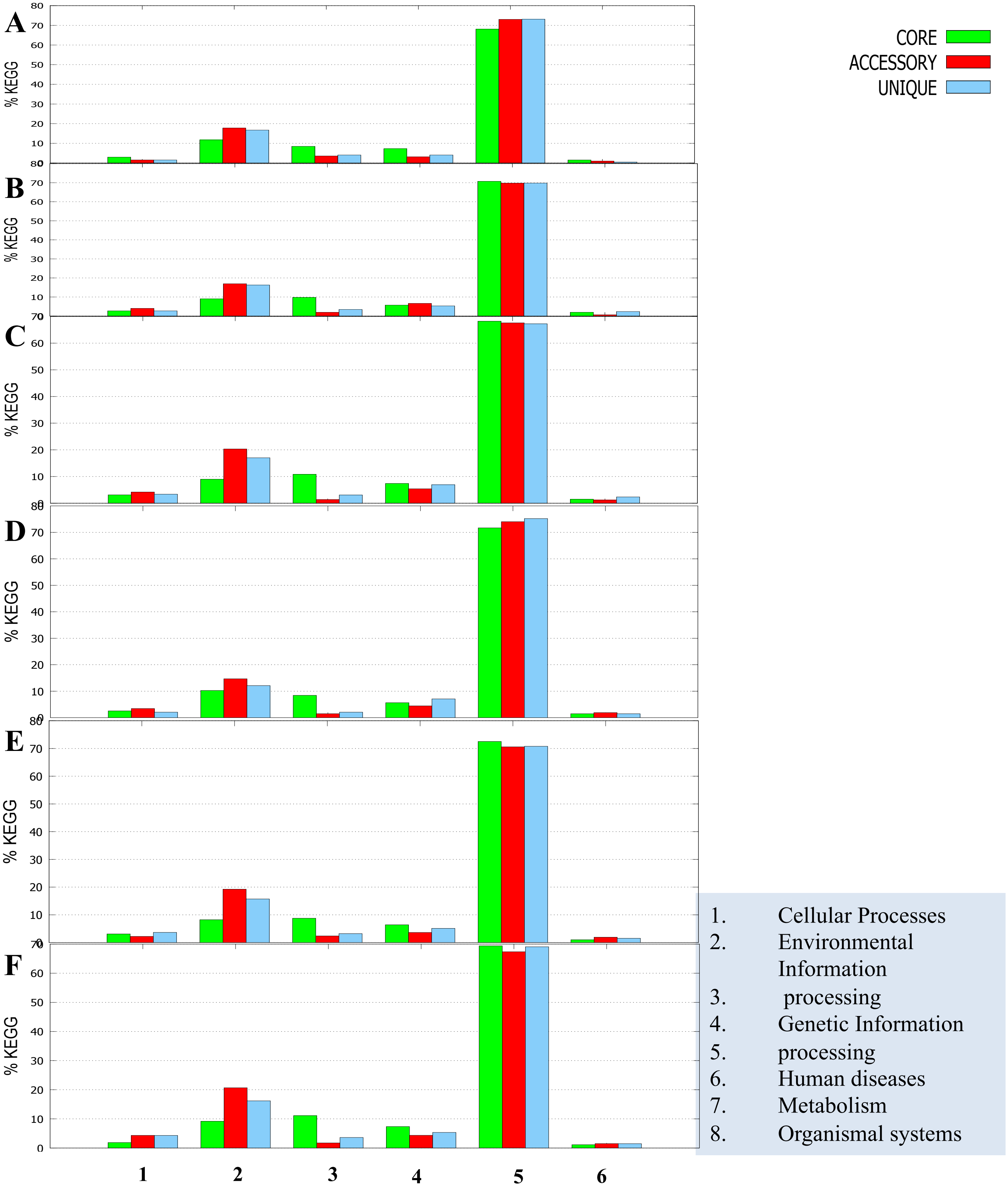

Supplementary Figure S2. Functional based KEGG distribution of core, accessory and unique genes present (A) Group I (B) Group II (C) Group (III) (D) Group V (E) Group VI (F) Group VII members of the genus *Roseomonas*

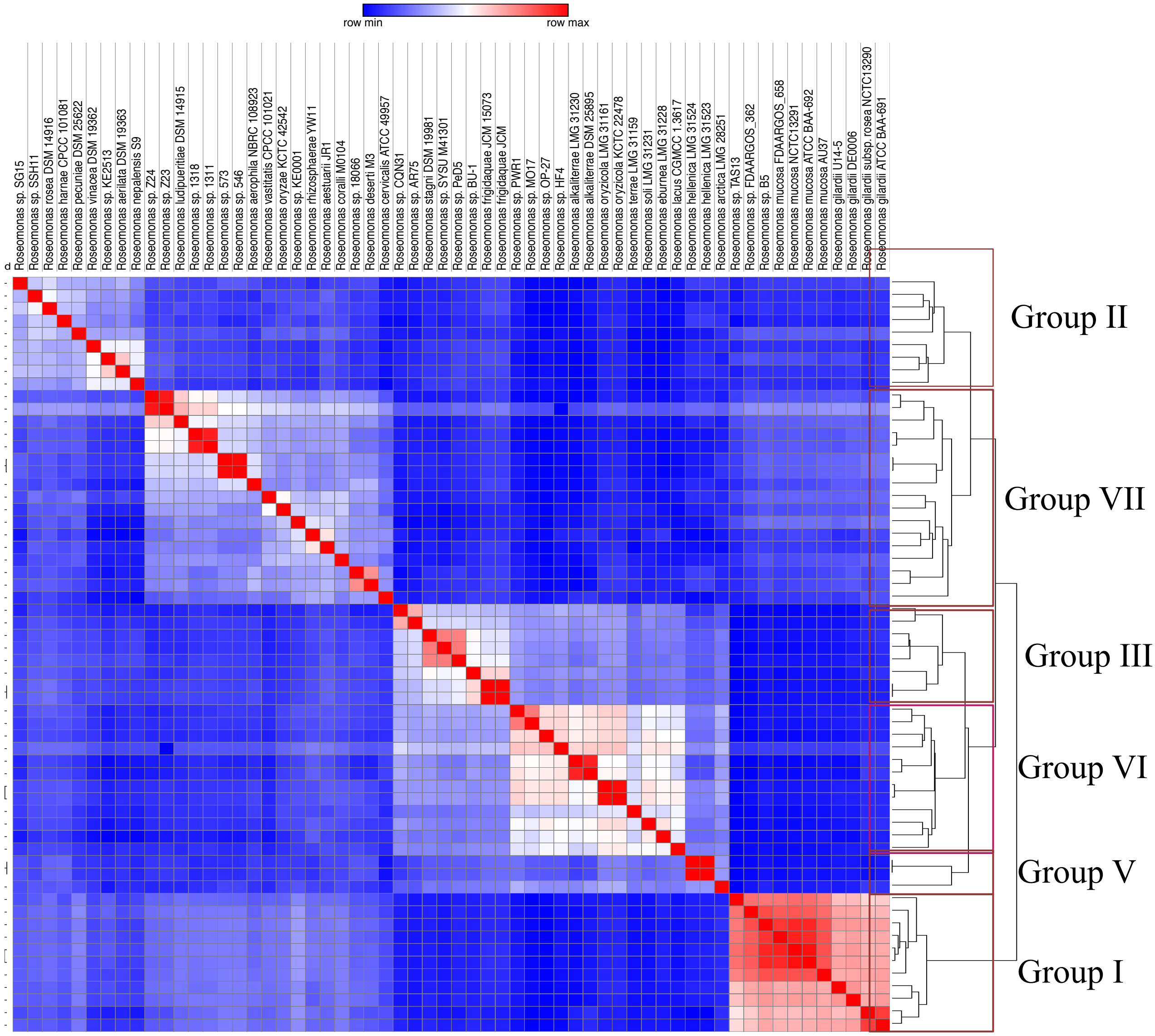

Supplementary Figure S3. Heatmap representing POCP values of the members of genus *Roseomonas* clustered with the complete h-clust method

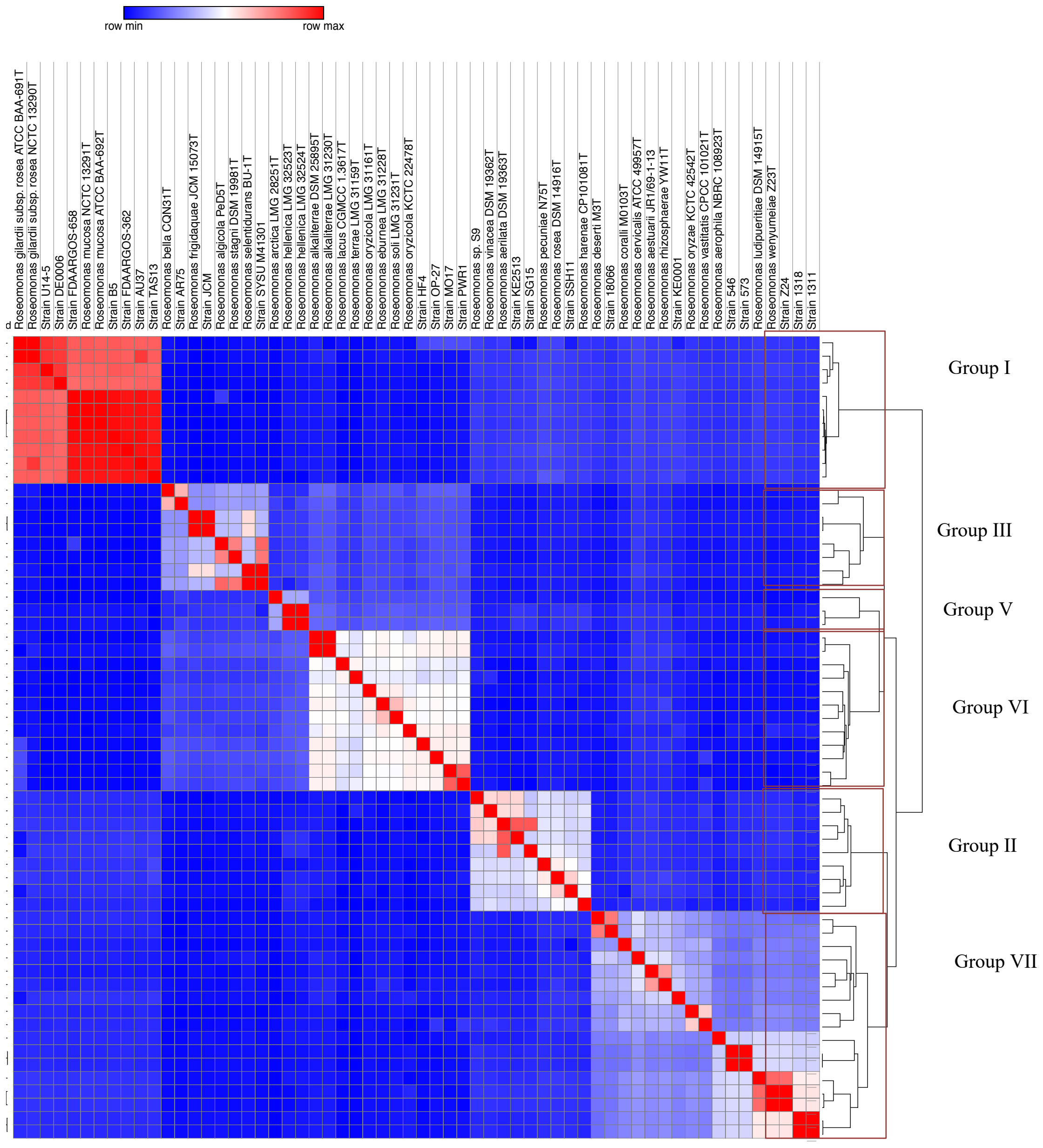

Supplementary Figure S4. Heatmap representing AAI values of the members of genus *Roseomonas* clustered with the complete h-clust method

*Table S1: The microorganisms used in this study, isolation sources and genome accession numbers

Taxa	Accession number	Source of Isolation
Acetobacter aceti NBRC 14818^T	BAMU01000001	Vinegar
Acetobacter ascendens LMG 1590 ^{T}	CP015164	Wine
Acetobacter cerevisiae LMG 1625 ^{T}	LHZA01000001	Beer
Acetobacter cibinongensis 4H-1 ^{T}	BAMV01000001	Fruit and curd
Acetobacter conturbans LMG 1627^T	WOSY01000001	Cider fermentation
Acetobacter estunensis LMG 1626^T	WOTH01000001	Food
Acetobacter fabarum KR	NCXK01000099	Cocoa beans
Acetobacter fallax LMG 1636^T	WOSX01000001	Cider frmentation
Acetobacter farinalis LMG 26772^T	WOTF01000001	Fermented rice flour
Acetobacter ghanensis LMG 23848T^T	LN609302	Fermented cocoa
Acetobacter indonesiensis 5H-1^T	BAMW01000001	Fermented products
Acetobacter lambici LMG 27439^T	WOTD01000001	Beer
Acetobacter lovaniensis DSM 4491^T	JACHIE010000001	Fruit
Acetobacter musti LMG 30640	WOTB01000001	Fruit
Acetobacter malorum LMG 1746^T	LHZC01000001	Apple
Acetobacter nitrogenifigens DSM 23921^T	AUBI01000001	Tea
Acetobacter oeni NBRC 105207	BJYG01000001	Spoiled red wine
Acetobacter okinawensis JCM 25146 ^{T}	BAJU01000127	Sugarcane
Acetobacter orientalis 21F-2^T	BAMX01000001	Fermented food
Acetobacter orleanensis JCM 7639^T	BAMY01000001	Vinegar
Acetobacter oryzifermentans SLV-7	CP022374	Fermented rice
Acetobacter oryzoeni B6^T	CP042808	Vinegar
Acetobacter papayae JCM 25143^T	BAIN01000177	Papaya
Acetobacter pasteurianus Ab3	CP012111	Vinegar
Acetobacter persici JCM 25330^T	BAJW01000236	Peach
Acetobacter pomorum BDGP5	CP023657	Gut Drosophila
Acetobacter sacchari TBRC 11175^T	JAFVMF010000090	Stem
Acetobacter sicerae LMG 1531^T	JAAABN010000001	Cider
Acetobacter senegalensis LMG 23690 ^{T}	LHZU01000001	Mango
Acetobacter suratthaniensis TBRC 1719	JAFVMG010000001	Fruit
Acetobacter syzygii 9H-2^T	BAMZ01000000	Fruit
Acetobacter thailandicus LMG 30826	WOSV01000001	Flower
Acetobacter tropicalis LMG 19825^T	LHZQ01000001	Coconut
Acidibrevibacterium fodinaquatile G45-3^T	CP029176	Acidic mine water
Acidicaldus organivorans DX-1	JPYW00000000	Geothermal site
Acidiphilium angustum ATCC 35903^T	JNJH01000001	Acid mine
Acidiphilium cryptum JF-5 ^{T}	009484	Coal mine sediment
Acidiphilium multivorum AIU301 ^{T}	015186	Mine drainage
Acidiphilium rubrum ATCC 35905^T	FTNE01000074	Mine drainage
Acidisphaera rubrifaciens HS-AP3^T	BANB01001239	Acidic environment
Acidocella aminolytica DSM 11237^T	FQVJ01000109	Mines
Acidocella facilis ATCC 35904 ^{T}	JHYG01000001	Mines
Acidomonas methanolica NBRC 104435	BAND01000546	Sludge
Asaia astilbis JCM 15831	BAJT01000049	fFowers
Asaia bogorensis NBRC 16594^T	AP014690	Flower of orchid
Asaia platycodi JCM 25414^T	BAKW01000035	Balloon flower
Asaia prunellae JCM 25354^T	BAJV00000000	*Prunella* sp. flowers
Asaia siamensis CCM 7132^T	BMCH01000001	Flowers
Ameyamaea chiangmaiensis LMG 27010	JABXXR00000000	Red ginger
Azospirillum brasilense SP7^T	VISK01000013	Root
Belnapia moabensis DSM 16746^T	JQKB00000000	Soil crust
Belnapia rosea CPCC 100156^T	FMZX00000000	Soil
Belnapia rosea CGMCC 1.10758^T	FMXZ01000105	Soil
Belnapia sp. F-4-1	KN676113	Soil
Belnapia sp. T18	JAETWB010000100	Desert biocrust surface
Belnapia sp. T6	JAEUXJ010000100	Desert Soil
Bombella apis MRM1	JADAQV010000001	Bee hive
Bombella intestini R-52487^T	JATM01000005	Bumblebee crop
Caldovatus sediminis CGMCC 1.16330^T	BMKS01000001	Hot-spring
Commensalibacter intestini A911^T	AGFR01000026	Drosophila gut
Crenalkalicoccus roseus YIM 78023 ^{T}	SJDM00000000	Alkaline hot spring
Dankookia rubra JCM 30602^T	SMSJ00000000	Sediment of stream
Endobacter medicaginis CECT 8088^T	JACHXV010000001	Nodules in an acidic soil
Entomobacter blattae G55GP^T	CP060244	Cockroaches
Gluconacetobacter asukensis LMG 27724^T	JABEQE010000001	
Gluconacetobacter aggeris LMG 27801	JABEQD010000001	Soil
Gluconacetobacter azotocaptans LMG 21311	JABEQF010000001	Plant
Gluconacetobacter diazotrophicus PA1 5^T	011365	Sugarcane roots
Gluconacetobacter entanii AV429	JABJWD010000072	Vinegar
Gluconacetobacter johannae LMG 21312	JABEQH010000001	Plant
Gluconacetobacter liquefaciens DSM 5603^T	QQAW01000001	Dried fruit
Gluconacetobacter sacchari LMG 19747	JABEQJ010000001	Plant
Gluconacetobacter takamatsuzukensis LMG 27800	JABEQK010000001	Soil
Gluconacetobacter tumulicola LMG 27725	JABEQL010000001	Environment
Gluconobacter albidus TMW2	CP014689	Water
Gluconobacter cadivus LMG 1744^T	JABCQI010000001	Rotting pear

Gluconobacter cerevisiae LMG 27748^T	JABCQO010000001	Beer
Gluconobacter cerinus NBRC 3267^T	BEWM01000001	cherry
Gluconobacter frateurii NBRC 3264^T	BEWN01000001	strawberry
Gluconobacter japonicus LMG 1373^T	LHZK00000000	Fruit
Gluconobacter kondonii NBRC 3266^T	BEWP01000001	Strawberry
Gluconobacter kanchanaburiensis NBRC 103587^T	BJVA01000001	Spoiled fruit
Gluconobacter morbifer G707^T	AGQV01000019	Gut of Drosophila
Gluconobacter oxydans H24	019396	Industry
Gluconobacter roseus NBRC 3990^T	BJLY01000001	Persimmon fruit
Gluconobacter sphaericus NBRC 12467^T	BJMK01000001	Fresh grape
Gluconobacter thailandicus HD924	CP043043	Wheat
Gluconobacter wancherniae NBRC 103581^T	BJUZ01000001	Seed
Granulibacter bethesdensis CGDNIH4^T	CP003182	Lymph node cultures
Humitalea rosea DSM 24525^T	QKYU00000000	Soil
Komagataeibacter cocois WE7^T	QEXL01000001	Contaminated coconut milk
Komagataeibacter diospyri MSKU15	BDLW01000001	Sapodilla fruit
Komagataeibacter europaeus NBRC 3261	BANI01000596	food
Komagataeibacter hansenii ATCC 23769	CM000920	Vinegar
Komagataeibacter intermedius AF2	JUFX02000232	Kombucha tea
Komagataeibacter kakiaceti DSM 24098^T	JACIJA010000001	Fruit vinegar
Komagataeibacter maltaceti LMG 1529^T	POTC01000001	Vinegar
Komagataeibacter medellinensis NBRC 3288	AP012159	Vinegar
Komagataeibacter melaceti AV382^T	QUWV01000092	Vinegar
Komagataeibacter melomenusus AV436^T	JABJWC010000001	Apple cider vinegar
Komagataeibacter nataicola RZS01	CP019875	(unspecified)
Komagataeibacter oboediens LMG 18849	NKTX01000100	Homemade vinegar
Komagataeibacter pomaceti T5K1	NOXG01000100	Vinegar fermentation sludge
Komagataeibacter rhaeticus LMG 22126^T	NKTY00000000	Vinegar
Komagataeibacter saccharivorans LMG 1582	NKTY01000073	Fruit fly
Komagataeibacter sucrofermentans LMG 18788^T	NKUA01000082	Vinegar
Komagataeibacter swingsii LMG 22125^T	NKUB01000099	Italian apple fruit
Komagataeibacter xylinus NBRC 13693	BANJ01000211	(unspecified)
Kozakia baliensis NBRC 16664^T	BJVW01000001	Palm brown sugar and ragi
Lichenicoccus roseus KEBCLARHB70R^T	VCDI01000001	*Cladonia arbuscula*
Lichenicola cladoniae PAMC 26569^T	CP053708	*Cladonia borealis*
Neoasaia chiangmaiensis NBRC 101099	BJXS01000001	Flower of red ginger
Neokomagataea tanensis AH13^T	CP032485	Candle bush
Neokomagataea thailandica NBRC 106555^T	BCZB01000001	Lantana plant
Nguyenibacter vanlangensis LMG 31431^T	JABXXP010000001	Rice
Oecophyllibacter saccharovorans Ha5^T	CP038143	Ant
Paracraurococcus ruber JCM 9931^T	SMOA00000000	Soil
Paracraurococcus ruber DSM 15382^T	NRSG01000001	Soil
Parasaccharibacter apium A29	LMYH01000016	Hindgut of bee
Rhodopila globiformis DSM 161^T	NHRY00000000	Sulphur spring
Rhodovarius lipocyclicus CCUG 44693^T	JAAABL00000000	Industrial hygiene control
Rhodovarius sp. CCP-6	SACL01000010	Water
Rhodovastum atsumiense DSM 21279	VWPK00000000	Paddy field
Roseicella frigidaeris DB1506^T	QLIX01000001	Air-conditioning system
Roseococcus suduntuyensis DSM 19979^T	JACIDJ010000001	Soda lake
Roseococcus thiosulfatophilus RB-3	JAFFQY010000011	marine cyanobacterial mat in hot spring
Roseomonas aeriglobus KER25-12^T	JAFHKN010000001	Air-conditioning system
Roseomonas aerilata DSM 19363^T	JONP00000000	Air sample
Roseomonas aerophila NBRC 108923^T	JACTVA00000000	Air
Roseomonas algicola PeD5^T	JAAIKB000000000	Green alga
Roseomonas alkaliterrae DSM 25895^T	JACIJE00000000	Geothermal soil
Roseomonas alkaliterrae LMG 3123^T	JAAEDJ010000001	Soil
Roseomonas arctica LMG 28251^T	JAAEDH010000001	Glacial Soil
Roseomonas bella CQN31^T	QGNA000000000	Lake sediment
Roseomonas cervicalis ATCC 49957^T	ADVL00000000	Blood
Roseomonas coralli M0104^T	SNVJ00000000	Gorgonian Coral
Roseomonas deserti M3^T	MLCO00000000	Oil contaminated desert soil
Roseomonas eburnea LMG 31228^T	JAAEDL010000001	Activated sludge
Roseomonas frigidaquae JCM 15073^T	JAAVTX00000000	Water cooling system
Roseomonas gilardii subsp. *rosea* ATCC BAA-691^T	JADY00000000	Blood
Roseomonas gilardii subsp. *rosea* NCTC13290^T	UGVO01000005	Blood
Roseomonas harenae CPCC 101081^T	WWDL01000001	Desert
Roseomonas hellenica LMG 31523^T	JAAGBB010000001	Plant root
Roseomonas lacus CGMCC 1.3617^T	BMKW00000000	Freshwater lake sediment
Roseomonas ludipueritiae DSM 14915^T	JACTUZ00000000	Indoor building material
Roseomonas mucosa NCTC 13291^T	UGVN00000000	Blood
Roseomonas mucosa ATCC BAA-692^T	JHWD01000001	Blood
Roseomonas oryzae KCTC 42542^T	VUKA00000000	Rhizosphere soil
Roseomonas oryzicola KCTC 22478^T	JAAVUP00000000	Rhizosphere of rice
Roseomonas oryzicola LMG 31161^T	JAAEDK010000001	Rhizosphere of rice
Roseomonas pecuniae N75^T	JACIJD00000000	Surface of coin
Roseomonas rhizosphaerae YW11^T	PDNU00000000	Soil
Roseomonas rosea DSM 14916^T	FQZF00000000	Indoor building material
Roseomonas selenitidurans BU-1^T	JAAVNE00000000	Urban soil
Roseomonas stagni DSM 19981^T	FOSQ00000000	Sediment of pond water
Roseomonas soli LMG 31231^T	JAAEDM00000000	Soil

Roseomonas terrae LMG 31159^T	JAAEDI010000001	Soil
Roseomonas vinacea DSM 19362^T	BO93DRAFT	Soil
Roseomonas vastitatis CPCC 101021 ^{T}	QXGS00000000	Badain desert
Roseomonas wenyumeiae Z23^T	RFLX00000000	Feces of Tibetan antelope
Roseomonas genomospecies 6	QOKW01000100	Breast incision
Roseomonas sp. JCM	JAATJR000000000	Water cooling system
Roseomonas sp. LMG 31524	JAAGBC010000001	Plant root
Roseomonas sp. U14-5	CP015583	Water
Roseomonas sp. DE0006	VEIX01000010	Environment
Roseomonas sp. S9.3B	RCZP00000000	Oil contaminated soil
Roseomonas sp. AU37	LLWF02000001	Peripheral intravenous catheter
Roseomonas sp. FDAARGOS_658	CP044114	Clinical isolate
Roseomonas sp. FDAARGOS_362	CP024588	Blood
Roseomonas sp. JR1/69-1-13	PDOA00000000	Marine water sample
Roseomonas sp. Z24	RAQU00000000	Feces of Tibetan antelope
Roseomonas sp. AR75	STGB00000000	Sediment
Roseomonas sp. HF4	STGD00000000	Sediment
Roseomonas sp. 18066	CACSJM010000001	Roots of beech
Roseomonas sp. B5	ALOX01000001	Soil
Roseomonas sp. 1311	JACTNF010000103	*Marmota himalayana*
Roseomonas sp. 1318	CP061091	*Marmota himalayana*
Roseomonas sp. TAS13	BDLP01000001	Activated sludge
Roseomonas sp. 573	JACTNG010000010	Tick
Roseomonas sp. 546	CP061177	Tick
Roseomonas sp. KE0001	RCVQ01000001	Automobile
Roseomonas sp. KE2513	RCVR01000001	Automobile
Roseomonas sp. MO17	JACADQ010000001	Paddy soil
Roseomonas sp. OP-27	JACADR010000001	Paddy soil
Roseomonas sp. PWR1	JAGIYZ010000001	Soil
Roseomonas sp. SG15	JAGIZA010000001	Soil
Roseomonas sp. SSH11	JAGIZB010000001	Soil
Roseomonas sp. SYSU M41301	JAERQN010000010	missing
Rubritepida flocculans DSM 14296 ^{T}	AUDH00000000	Hot spring
Saccharibacter floricola DSM 15669^T	KB899333	pollen
Siccirubricoccus deserti CGMCC 1.15936^T	BMKH0100000	Desert sample
Siccirubricoccus phaeus 1-3^T	SRXO01000001	Oil soil
Swaminathania salitolerans NBRC 104436	BJVC01000001	Plant
Swingsia samuiensis AH83^T	CP038141	Plant
Tanticharoenia sakaeratensis NBRC 103193^T	BALE01000094	Soil
Stella humosa DSM 5900^T	RJKX01000011	Soil

Table S2. Ninety two genes considered for the construction of phylogenomic tree UBCG tool (Na et al., 2018)

Gene	Function	Genes	Function
alaS	Alanine-tRNA ligase	*rpmA*	50S ribosomal protein L27
argS	Arginine-tRNA ligase	*rpmC*	50S ribosomal protein L29
aspS	Aspartate-tRNA ligase	*rpmI*	50S ribosomal protein L35
cgtA	GTPase ObgE/CgtA	*rpoA*	DNA-directed RNA polymerase subunit alpha
coaE	Dephospho-CoA kinase	*rpoB*	DNA-directed RNA polymerase subunit beta
cysS	Cysteine-tRNA ligase	*rpoC*	DNA-directed RNA polymerase subunit beta'
dnaA	Chromosomal replication initiator protein DnaA	*rpsB*	30S ribosomal protein S2
dnaG	DNA primase	*rpsC*	30S ribosomal protein S3
dnaX	DNA polymerase III subunit gamma	*rpsD*	30S ribosomal protein S4
engA	GTPase Der	*rpsE*	30S ribosomal protein S5
ffh	Signal recognition particle protein	*rpsF*	30S ribosomal protein S6
fmt	Methionyl-tRNA formyltransferase	*rpsG*	30S ribosomal protein S7
frr	Ribosome-recycling factor	*rpsH*	30S ribosomal protein S8
fts	Signal recognition particle receptor FtsY	*rpsI*	30S ribosomal protein S9
gmk	Guanylate kinase	*rpsJ*	30S ribosomal protein S10
hisS	Histidine-tRNA ligase	*rpsK*	30S ribosomal protein S11
ileS	Isoleucine-tRNA ligase 1	*rpsL*	30S ribosomal protein S12
infB	Translation initiation factor IF-2	*rpsM*	30S ribosomal protein S13
infC	Translation initiation factor IF-3	*rpsO*	30S ribosomal protein S15
ksgA	Ribosomal RNA small subunit methyltransferase A	*rpsP*	30S ribosomal protein S16
lepA	Elongation factor 4	*rpsQ*	30S ribosomal protein S17
leuS	Leucine-tRNA ligase	*rpsR*	30S ribosomal protein S18
ligA	DNA ligase	*rpsS*	30S ribosomal protein S19
nusA	Transcription termination/antitermination protein NusA	*rpsT*	30S ribosomal protein S20
nusG	Transcription termination/antitermination protein NusG	*secA*	Protein translocase subunit SecA
pgk	Phosphoglycerate kinase	*secG*	Protein-export membrane protein SecG
pheS	Phenylalanine-tRNA ligase alpha subunit	*secY*	Protein translocase subunit SecY
pheT	Phenylalanine-tRNA ligase beta subunit	*serS*	Serine-tRNA ligase
prfA	Peptide chain release factor 1	*smpB*	SsrA-binding protein
pyrG	CTP synthase	*tig*	Trigger factor
recA	DNA recombination and repair protein	*tilS*	tRNA(Ile)-lysidine synthase
rbfA	30S ribosome-binding factor	*truB*	tRNA pseudouridine synthase B
rnc	Ribonuclease 3	*tsaD*	tRNA N6-adenosine threonylcarbamoyltransferase
rplA	50S ribosomal protein L1	*tsf*	Elongation factor Ts
rplB	50S ribosomal protein L2	*uvrB*	UvrABC system protein B
rplC	50S ribosomal protein L3	*ybeY*	Endoribonuclease YbeY
rplD	50S ribosomal protein L4	*ychF*	Ribosome-binding ATPase YchF
rplE	50S ribosomal protein L5		
rplF	50S ribosomal protein L6		
rplI	50S ribosomal protein L9		
rplJ	50S ribosomal protein L10		
rplK	50S ribosomal protein L11		
rplL	50S ribosomal protein L7/L12		
rplM	50S ribosomal protein L13		
rplN	50S ribosomal protein L14		
rplO	50S ribosomal protein L15		
rplP	50S ribosomal protein L16		
rplQ	50S ribosomal protein L17		
rplR	50S ribosomal protein L18		
rplS	50S ribosomal protein L19		
rplT	50S ribosomal protein L20		
rplU	50S ribosomal protein L21		
rplV	50S ribosomal protein L22		
rplW	50S ribosomal protein L23		
rplX	50S ribosomal protein L24		

Table S4: Bacterial Pan Genome Analysis (BPGA) of the members of the genus *Roseomonas* (Group-wise)

Taxa	No. of core genes	No. of accessory genes	No. of unique genes	No. of exclusively absent genes
**(n=60)**				
R. gilardii subsp. *rosea* ATCC BAA-691^T	958	3169	94	0
R. gilardii subsp. *rosea* NCTC 13290^T	958	2766	22	3
R. mucosa ATCC BAA-692^T	958	3447	20	0
R. mucosa NCTC 13291^T	958	3452	13	1
R. sp. DE0006	958	3352	315	0
R. sp. U14-5	958	3504	408	0
R. sp. AU37	958	3269	143	0
R. sp. B5	958	3357	17	0
R. sp. TAS13	958	3578	308	3
R. sp. FDAARGOS_658	958	3443	62	0
R. sp. FDAARGOS_362	958	3382	142	0
R. rosea DSM 14916^T	958	3539	419	1
R. aerilata DSM 19363^T	958	4181	770	0
R. pecuniae N75^T	958	3027	577	1
R. vinacea DSM 19362^T	958	4059	938	0
R. harenae CPCC 101081^T	958	3340	736	1
R. sp. SSH11	958	3275	574	0
R. sp. KE2513	958	4239	690	1
R. sp. S9.3B	958	4012	1220	1
R. sp. SG15	958	3568	831	4
R. stagni DSM 19981^T	958	4380	345	2
R. algicola PeD5^T	958	4607	393	0
R. bella CQN31^T	958	3869	523	2
R. frigidaquae JCM 15073^T	958	4628	1	0
R. selenitidurans BU-1^T	958	3707	698	1
R. sp. AR75	958	4068	741	0
R. sp. SYSU M4 1301	958	4632	443	0
R. sp. JCM	958	4629	0	0
R. arctica LMG 28251^T	958	2497	742	15
'R. hellenica' LMG 31523^T	958	5742	0	0
R. sp. LMG 31524	958	5743	0	0
R. oryzicola KCTC 22478^T	958	3987	13	0
R. oryzicola LMG 31161^T	958	4022	53	0
R. alkaliterrae DSM 25895^T	958	2970	44	0
R. alkaliterrae LMG 31230^T	958	3064	153	3
R. lacus CGMCC 1.3617^T	958	4105	878	0
R. eburnea LMG 31228^T	958	3723	695	0
R. terrae LMG 31159^T	958	3489	959	2
R. soli LMG 31523^T	958	3462	606	1
R. sp. OP-27	958	3608	620	0
R. sp. PWR1	958	3431	314	0
R. sp. HF4	958	3537	505	1
R. sp. MO17	958	3557	438	1
R. deserti M3^T	958	4157	639	2
R. aerophila NBRC 108923^T	958	3319	735	2
R. cervicalis ATCC 49957^T	958	2817	750	20
R. coralli M0104^T	958	2990	677	2
R. ludipueritiae DSM 14915^T	958	3606	545	2
R. oryzae KCTC 42542^T	958	2922	465	1
R. rhizosphaerae YW11^T	958	2941	370	4
R. vastitatis CPCC 101021^T	958	3175	770	0
R. wenyumeia Z23^T	958	4375	82	0
R. sp. JR1/69-1-13	958	3353	435	0
R. sp. 18066	958	4117	581	2
R. sp. KE0001	958	2648	460	3
R. sp. 546	958	3453	24	0
R. sp. 573	958	3457	26	0
R. sp. 1311	958	3344	70	0
R. sp. 1318	958	3304	112	0
R. sp. Z24	958	4359	116	0
**Pan genome analysis of Group I clade (n=11)**				
R. gilardii subsp. *rosea* ATCC BAA-691^T	3152	883	153	3
R. gilardii subsp. *rosea* NCTC 13290^T	3152	558	20	147
R. mucosa NCTC 13291^T	3152	1236	9	1
R. mucosa ATCC BAA-692^T	3152	1230	21	0
R. sp. DE0006	3152	986	465	45
R. sp. U14-5	3152	1128	564	11
R. sp. AU37	3152	1025	164	5
R. sp. B5	3152	1211	388	24
R. sp. TAS13	3152	1201	83	3
R. sp. FDAARGOS_658	3152	1120	174	8
R. sp. FDAARGOS_362	3152	883	153	3
**Pan genome analysis of Group II clade (n=9)**				
R. rosea DSM 14916^T	2194	2042	652	25
R. aerilata DSM 19363^T	2194	2774	879	4
R. pecuniae N75^T	2194	1585	742	130
R. vinacea DSM 19362^T	2194	2662	1062	11
R. harenae CPCC 101081^T	2194	1750	1044	88
R. sp. SSH11	2194	1869	712	37
R. sp. KE2513	2194	2818	816	19
R. sp. S9.3B	2194	2557	1380	19
R. sp. SG15	2194	2019	1116	76
**Pan genome analysis of Group III clade (n=8)**				
R. stagni DSM 19981^T	1573	3643	450	8
R. algicola PeD5^T	1573	3861	495	0
R. bella CQN31^T	1573	2242	1512	56
R. frigidaquae JCM 15073^T	1573	3979	1	0

R. selenitidurans BU-1^{T}	1573	2865	898	22
R. sp. AR75	1573	584	2199	1099
R. sp. SYSU M4 1301	1573	3873	565	2
R. sp. JCM	1573	3979	0	0
**Pan genome analysis of Group V clade (n=3)**				
R. arctica LMG 28251^{T}	2927	1	1242	3700
'R. hellenica' LMG 31523^{T}	2927	3701	0	0
R. sp. LMG 31524	2927	3700	0	1
**Pan genome analysis of Group VI clade (n=12)**				
R. lacus CGMCC 1.3617^{T}	2448	2247	1199	15
R. oryzicola KCTC 22478^{T}	2448	2464	15	1
R. oryzicola LMG 31161^{T}	2448	2478	53	2
R. alkaliterrae DSM 25895^{T}	2448	1454	53	2
R. alkaliterrae LMG 31230	2448	1497	148	4
R. eburnea LMG 31228^{T}	2448	1967	922	14
R. terrae LMG 31159^{T}	2448	1655	1262	116
R. soli LMG 31523^{T}	2448	1836	702	23
R. sp.OP-27	2448	1953	682	31
R. sp. PWR1	2448	1853	385	9
R. sp. HF4	2448	1902	616	29
R. sp. MO17	2448	1920	466	8
**Pan genome analysis of Group VII clade (n=17)**				
R. cervicalis ATCC 49957^{T}	1591	1982	870	78
R. deserti M3^{T}	1591	3420	691	5
R. aerophila NBRC 108923^{T}	1591	2471	928	15
R. coralli M0104^{T}	1591	2159	858	25
R. ludipueritiae DSM 14915^{T}	1591	2833	647	6
R. oryzae KCTC 42542^{T}	1591	2190	548	17
R. rhizosphaerae YW11^{T}	1591	2139	525	26
R. vastitatis CPCC 101021^{T}	1591	2400	890	4
R. wenyumeia Z23^{T}	1591	3699	94	0
R. sp. JR1/69-1-13	1591	2610	516	3
R. sp. 18066	1591	3353	672	4
R. sp. KE0001	1591	1892	572	27
R. sp. 546	1591	2804	24	0
R. sp. 573	1591	2808	25	0
R. sp. 1311	1591	2686	74	0
R. sp. 1318	1591	2650	116	0
R. sp. Z24	1591	3668	118	2
